# A low-noise photonic heterodyne synthesizer and its application to millimeter-wave radar

**DOI:** 10.1038/s41467-021-24637-0

**Published:** 2021-07-20

**Authors:** Eric A. Kittlaus, Danny Eliyahu, Setareh Ganji, Skip Williams, Andrey B. Matsko, Ken B. Cooper, Siamak Forouhar

**Affiliations:** 1 Jet Propulsion Laboratory, California Institute of Technology, Pasadena, CA USA; 2 OEwaves Inc., Pasadena, CA USA

**Keywords:** Microwave photonics, Photonic devices

## Abstract

Microwave photonics offers transformative capabilities for ultra-wideband electronic signal processing and frequency synthesis with record-low phase noise levels. Despite the intrinsic bandwidth of optical systems operating at ~200 THz carrier frequencies, many schemes for high-performance photonics-based microwave generation lack broadband tunability, and experience tradeoffs between noise level, complexity, and frequency. An alternative approach uses direct frequency down-mixing of two tunable semiconductor lasers on a fast photodiode. This form of optical heterodyning is frequency-agile, but experimental realizations have been hindered by the relatively high noise of free-running lasers. Here, we demonstrate a heterodyne synthesizer based on ultralow-noise self-injection-locked lasers, enabling highly-coherent, photonics-based microwave and millimeter-wave generation. Continuously-tunable operation is realized from 1-104 GHz, with constant phase noise of -109 dBc/Hz at 100 kHz offset from carrier. To explore its practical utility, we leverage this photonic source as the local oscillator within a 95-GHz frequency-modulated continuous wave (FMCW) radar. Through field testing, we observe dramatic reduction in phase-noise-related Doppler and ranging artifacts as compared to the radar’s existing electronic synthesizer. These results establish strong potential for coherent heterodyne millimeter-wave generation, opening the door to a variety of future applications including high-dynamic range remote sensing, wideband wireless communications, and THz spectroscopy.

## Abstract (web summary)

Photonics-based radars offer intriguing potential but face tradeoffs in tunability, complexity, and noise. Here the authors present microwave generation in a photonics platform by heterodyning of two low-noise, self-injection-locked lasers, and demonstrate its advantages in an FMCW radar system.

## Introduction

High-performance microwave oscillators are key components for wide-ranging applications including communications^1–5^, metrology^6–8^, navigation^9^, security^10,11^, and spectrometry^12,13^. Beyond these traditional systems, microwave sources form the backbone of many scientific instruments such as heterodyne spectrometers^14–16^ and spaceborne atmospheric radars^17,18^. Due to both rapidly increasing spectral utilization for communications and new scientific opportunities at higher frequency bands, there is a pressing need for systems that can operate directly at high frequencies and with ultrawide bandwidth^19–21^. At the same time, next-generation microwave oscillators will have increasingly stringent requirements on spectral purity (phase noise level), which affects the achievable information capacity of communications links, as well as the dynamic range of microwave sensors and radars^22–24^. However, existing all-electronic systems have limited signal bandwidth and experience rapid performance degradation as carrier frequency is increased^19,25–27^.

By comparison, photonics-based approaches to radiofrequency (RF) signal synthesis may leverage the intrinsic broad bandwidth of optical systems and operate directly at high frequencies^26,28^. Experimental demonstrations have harnessed the superb stability of optical oscillator technologies to achieve low-noise microwave generation within a variety of platforms, including femtosecond lasers^29,30^, electro-optic frequency dividers^31^, soliton frequency combs^32–37^, Brillouin lasers^38–43^, and opto-electronic oscillators^44–50^. Each of these approaches has achieved phase noise performance comparable or superior to conventional electronics, demonstrating strong potential for photonics-based synthesizers within future microwave systems.

However, existing schemes for photonics-based microwave generation tend to be some combination of complex, bulky, or limited to specific operation frequencies or conditions. For example, opto-electronic oscillators enable impressive performance-to-size ratio^51^, but are typically not frequency-agile, and require active, low-loss electronic and electro-optic components, leading to decreased performance when scaled to higher frequencies. Soliton frequency combs represent one of the leading technologies for all-optical frequency synthesis^33,35–37,52^, but are fixed in repetition rate, and hence frequency, by the optical cavity geometry. Accessing the intrinsic bandwidth of photonics to enable widely tunable microwave synthesis at frequencies and performance levels beyond conventional electronic synthesizers remains a key goal^53,54^.

THz-range frequency tuning is routinely accomplished with optical lasers operating at carrier frequencies in the 100s of THz. As a result, it is straightforward to implement frequency-agile microwave synthesis through direct down-mixing of two such sources on a fast photodiode^55–58^. Experimental demonstrations have achieved operation from MHz to THz frequencies using this heterodyne approach^59–62^, and have shown potential for chip-based integration^62^. Laser heterodyning is attractive for its inherent simplicity; it requires relatively few components, and permits continuously tunable frequency synthesis with frequency-independent performance. However, the high noise of typical free-running lasers means that the phase noise level of heterodyne sources is typically several orders of magnitude worse than those achieved with existing synthesizers, particularly at low (GHz) frequencies. As a result, practical uses have been mainly restricted to THz frequencies where no agile electronic sources are available^60,63^.

In this article, we report widely tunable, low-noise millimeter-wave generation based on direct down-mixing of miniaturized external cavity lasers, and utilize this approach to significantly improve the dynamic range of a 95 GHz Doppler radar. The lasers used for heterodyne synthesis harness passive self-injection-locking to ultrahigh-quality factor (*Q* ~ 10^9^) microresonators, enabling highly coherent, tunable laser oscillation in a compact form factor. Through down-mixing of two such lasers from optical (~193 THz) to radio frequencies, microwave synthesis is achieved from 1 to 104 GHz with carrier-frequency-independent phase noise of −109 dBc/Hz at 100 kHz offset. The total power consumption of this system is <6 W, and the output power is >100 μW across the entire frequency range. Direct voltage-controlled frequency modulation is also implemented. As a demonstration of its practical utility, we use this source as the local oscillator in a 95-GHz continuous-wave radar, and show dramatic reduction in phase-noise-related ranging and Doppler artifacts as compared to using the radar’s existing silicon CMOS W-band synthesizer. These tests establish the feasibility of continuous-wave lasers for low-noise signal generation in future photonics-based radars. More generally, the realization of an ultralow-noise photonic heterodyne synthesizer opens the door to tunable, coherent millimeter- and THz-wave generation for a wide range of radar, communications, and precision sensing applications.

## Results

### Tunable microwave generation via photo-mixing

**Fig. 1:**
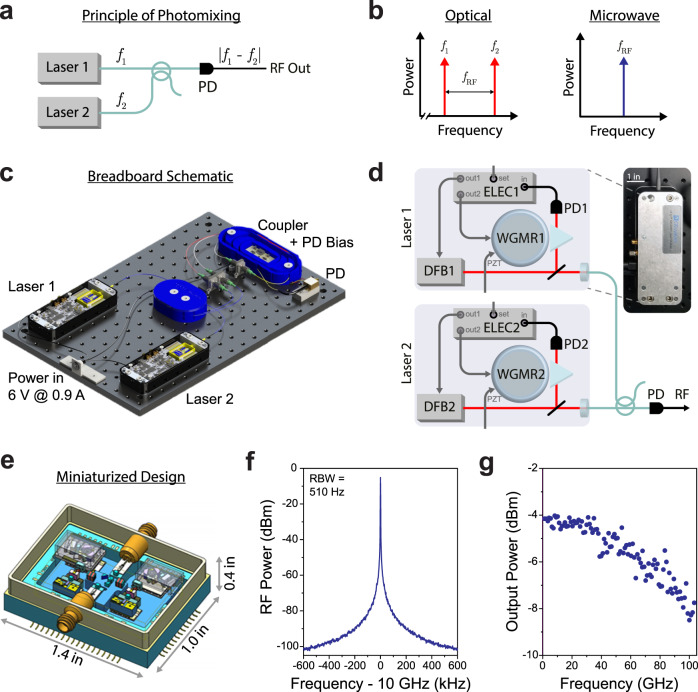
Microwave and millimeter-wave generation based on heterodyning of self-injection-locked lasers. **a** diagrams the photomixing (heterodyne) process. Two lasers at optical frequencies *f*_1_ and *f*_2_ are combined on a fast photodiode (PD). The resulting interference beat-note is converted to a RF signal at *f*_RF_ = ∣*f*_1_ − *f*_2_∣. The output frequency is varied by tuning of either laser. **b** depicts this process in the frequency domain for both input optical and output microwave tones. **c** shows an artistic representation of the heterodyne synthesizer on an optical breadboard. **d** diagrams the microwave source in greater detail, showing the internal components of the laser oscillators. Each consists of a distributed feedback laser (DFB1; DFB2) that is prism-coupled to a high-finesse whispering-gallery-mode microresonator (WGMR1; WGMR2). A small amount of back-reflection from the resonator into the laser diode leads to passive self-injection-locking to the resonator modes, and dramatic reduction in laser linewidth, through the external cavity feedback effect. Transmitted light is incident on a photodiode (PD1; PD2) for monitoring, and both the microresonator and laser diode are temperature-controlled by internal control electronics (ELEC1; ELEC2). The laser output frequency is controlled by varying the temperature set-point, and each WGMR can also be strain-tuned by applying an external voltage to a piezoelectric element (PZT). Light from each laser is fiber-coupled with a collimating lens. The two optical signals are combined via a fiber coupler on a fast photodiode (PD) for heterodyne synthesis. A photograph of one packaged laser is shown on the right. **e** shows an illustration of a miniaturized heterodyne synthesizer package under development, consisting of a pair of self-injection-locked lasers, fast photodiodes, and 1.0 mm coaxial outputs. **f** plots the output microwave spectrum when the oscillator is tuned to *f*_RF_ = 10 GHz as detected by a radiofrequency spectrum analyzer, with a resolution bandwidth of 510 Hz. **g** plots the measured output power as the oscillator frequency is varied between 1 and 104 GHz, showing a modest rolloff due to the frequency response of the photodiode.

Generally speaking, photonics-based microwave generation is based on down-mixing two or more optical signals to microwave frequencies through heterodyning. One simple implementation of this process is diagrammed in Fig. 1a–b; two lasers operating at optical frequencies *f*_1_ and *f*_2_ are combined and incident on a fast photodiode, resulting in an output signal at ∣*f*_1_ − *f*_2_∣. This output frequency is directly tunable by changing either *f*_1_ or *f*_2_, provided that the photodiode response bandwidth exceeds the target microwave frequency.

This form of direct laser heterodyning is an attractive approach for microwave synthesis due to its wideband tunability and intrinsic simplicity. Compared to other techniques for photonics-based microwave generation, such as frequency combs, cascaded Brillouin lasers, and opto-electronic oscillators, the output frequency can be continuously varied over extremely wide ranges with consistent performance. This tuning range is limited only by that of the source lasers—which can easily be many THz—and by the bandwidth of the photodetector used for down-mixing, which is common to all photonics-based microwave sources.

However, as compared to other microwave-photonic (RF-photonic) oscillator technologies that have produced world-record phase noise levels, heterodyne synthesizers based on two discrete laser oscillators usually exhibit poor noise characteristics. This is due to the relatively mediocre noise performance of typical free-running semiconductor lasers (at frequencies >100 THz) relative to more complex hybrid oscillator technologies designed for operation at microwave frequencies. As a result, heterodyne synthesizers have generally been restricted to incoherent applications or THz frequencies where suitable microwave sources are not available.

To overcome this limitation, we developed a heterodyne synthesizer based on ultralow-noise external cavity lasers. These lasers implement self-injecting-locking of a commercial distributed feedback (DFB) laser diode to an ultrahigh-quality-factor (*Q* = 10^9^) micromachined whispering gallery mode resonator (WGMR)^64–66^. The long energy storage times and stable operation of this cavity configuration enable ultralow-noise laser operation with Hz-level instantaneous linewidths, while the resonator’s microscale mass and volume make the laser system inherently insensitive to acceleration and vibration^64^, critical to implementation in field-deployable systems while maintaining low oscillator phase noise^67^. Precise thermal control, necessary both for laser stability and to implement frequency tuning, is also simplified by implementation of the laser, coupling optics, and WGMR in a miniaturized form-factor. The combined self-injection-locked laser modules and control electronics are integrated in a vibration-insensitive package to ensure robust and turnkey operation. Altogether, these attributes are attractive for a variety of practical microwave photonics applications.

Leveraging the excellent performance characteristics of self-injection-locked lasers, we construct the breadboard microwave synthesizer diagrammed in Fig. 1c–d. This apparatus consists of two self-injection-locked lasers manufactured through a commercial process (OEwaves HI-Q OE4030) and selected for ultra-low-noise operation, and a high-frequency photodiode (Finisar XPDV4121R; bandwidth >100 GHz). Each laser is packaged with a microscale WGM resonator, and is fiber-coupled using a collimating lens (Fig. 1d). Both lasers, as well as the photodiode bias circuit, are driven by a single DC power supply, with a total power consumption of <6 W. Of this power budget, around 5 W is used for the laser control subsystem and thermal management, while <1 W is required for electrical pumping of the two lasers. Importantly, all of the sub-components comprising the synthesizer are amenable to miniaturized packaging; Fig. 1e depicts the design of such a device currently under development.

Through operation of the heterodyne synthesizer, one laser is set to a fixed frequency of 193.3918 THz (1550.182 nm), while the other is varied between 193.3918 and 193.496 THz. Tunability is achieved through calibrated thermal control of the DFB laser module and WGM resonator via a USB interface; to achieve wide (>100 GHz) frequency agility, separate thermal control is implemented for each due to the distinct tuning curves of the DFB laser and resonator modes. The sweep rate for continuous thermal tuning is about 1 GHz/s, while for large frequency hops >10–100 GHz around 30–60 s is required to reach thermal equilibrium and self-injection-locked operation. The thermal tuning resolution is 5 MHz, and the precision/repeatability is around 20 MHz, limited by the current calibration scheme.

Alternately, two external SMA ports implement direct voltage-controlled frequency tuning: the first allows for thermal fine-tuning, while the second permits strain tuning via a piezoelectric transducer (PZT) laminated on the WGM resonator. Through elasto-optic modulation of the resonator modes, this PZT port enables frequency variation over a range of >±100 MHz with a bandwidth of >100 kHz; the measured PZT frequency shift is around 15 MHz/V (see Supplementary Note 4 for details). This same mechanism permits voltage-controlled frequency modulation of the optical (and hence output RF) signal, which can be applied to implement voltage-controlled waveform generation and chirp synthesis (see Supplementary Note 4). We operate the synthesizer in a free-running mode; however, frequency locking may be readily implemented through external referencing if necessary for specific applications.

This source allows continuously tunable RF signal generation from <1 to 104 GHz, limited by the photodiode operation bandwidth. Through operation, each self-injection-locked laser outputs 17 mW continuous-wave power, and the two are combined through a 50:50 fiber coupler onto the fast photodiode, resulting in optical powers of *P*_1_ = *P*_2_ = 8.5 mW for the two tones. The beat-note between these two signals results in a time-harmonic photocurrent ${I}_{{{\Omega }}} \sim 2\Re \sqrt{{P}_{1}{P}_{2}}\cos {{\Omega }}t$ proportional to the detector responsivity *ℜ*. The corresponding output RF power is ${P}_{{{\Omega }}}=<{I}_{{{\Omega }}}^{2}> {R}_{{\rm{o}}}{\left|{H}_{{\rm{PD}}}\right|}^{2},$ where *R*_o_ = 50 Ω is the output resistance and *H*_PD_ is the photodiode circuit function (≈0.5 at low frequencies due to impedance matching)^25^. At low frequencies, measured output RF power *P*_Ω_ = 400 μW implies that *ℜ* = 0.47 A/W. Experimental data show a representative spectrum analyzer trace for an operating frequency of 10 GHz (Fig. 1f), and output power of the heterodyne synthesizer as its frequency is varied between 1 and 104 GHz (Fig. 1g) as measured on a fast spectrum analyzer (Anritsu MS2760A). The synthesizer produces output powers >100 μW over the entire frequency range, with a modest frequency rolloff due to the photodiode response.

### Phase noise characterization

Though the heterodyne process, the phase noise of the output microwave signal is directly set by the sum of the phase noise powers of the two independent lasers. This resulting noise level is expected to be independent of operation frequency since it arises from a down-mixing process, rather than frequency multiplication as in electronic synthesizers. Here, we experimentally characterize the phase noise level of the heterodyne source and its constituent lasers, revealing low-noise operation from 1 to 104 GHz.

Direct all-electronic phase noise measurements at high frequencies are nontrivial due to the lack of low-noise reference oscillators, and increased noise and losses through mixing and amplification steps. Therefore, to perform phase noise measurements of the heterodyne synthesizer across the microwave W band (75–110 GHz), we construct a microwave-photonic frequency discriminator based on a low-loss fiber-optic delay line. This approach is similar to a traditional microwave frequency discriminator^68^, and allows sensitive noise measurements without the need for an external frequency reference.

**Fig. 2:**
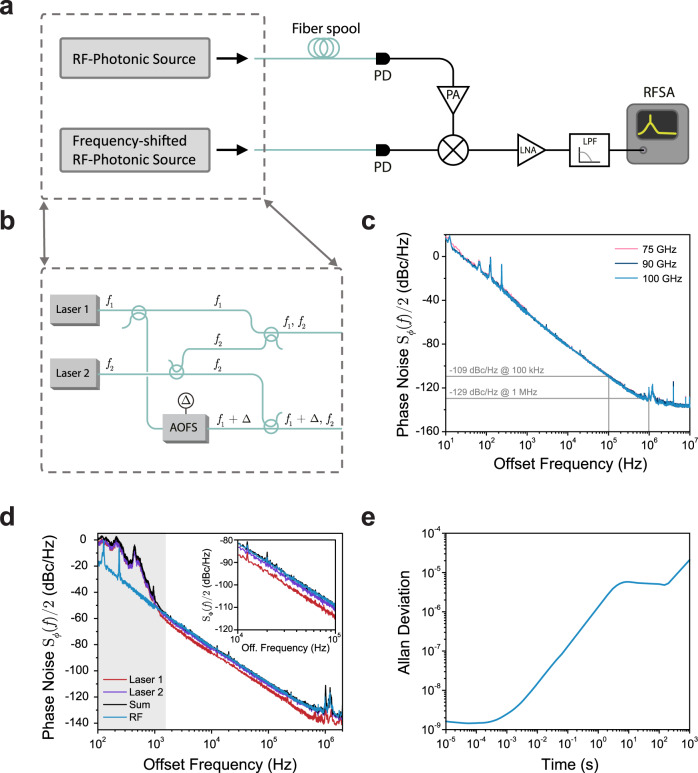
Phase noise measurements of the heterodyne source. **a** diagrams the basic concept of the frequency discriminator experiment. Two copies of the millimeter-wave signal are synthesized by splitting the two lasers, but one is frequency-shifted by Δ = 100 MHz using an optical single-sideband modulator driven by an external low-noise microwave source. A long fiber-optic delay line produces a time-delay *τ* between the two signals, partially de-correlating them. Each is then incident on a fast photodiode (PD). One signal is amplified to bias a W-band mixer, and the two signals are mixed down to Δ, before being passed through a low-noise amplifier (LNA) and low-pass filter. Phase (frequency) fluctuations over the time delay *τ* are converted into a noise pedestal around Δ which reflects the phase noise content of the microwave source with a well-defined transfer function. **b** shows how two pairs of frequency-detuned optical signals are synthesized from the same two lasers. One optical tone is shifted by +Δ using an acousto-optic frequency shifter (AOFS). **c** plots the measured single-sideband phase noise *S*_*ϕ*_(*f*)/2 for output frequencies of 75, 90, and 100 GHz. Because the noise level of the source lasers does not change appreciably as they are tuned, the phase noise of the down-mixed microwave signal is constant with operating frequency. **d** compares the measured phase noise at 100 GHz with the measured phase noise of each laser. As expected, the phase noise of the microwave source matches the sum of the phase noise of the two lasers. At offset frequencies <1 kHz (shaded gray), additional fiber-optic-induced noise from environmental vibrations prohibits accurate measurement of the laser phase noise. **e** plots the Allan deviation of the free-running source at a center frequency of 100 GHz. Below averaging times of 0.1 s, the Allan deviation is calculated from the phase noise distribution, while above 0.1 s it is calculated from frequency counter measurements.

The phase noise measurement apparatus is sketched in Fig. 2a–b. Two copies of the microwave-photonic source are synthesized from the same two lasers using fiber-optic directional couplers. A frequency-shift is implemented in one of the two copies by passing one of the optical tones through an acousto-optic frequency shifter (AOFS) that acts as an optical single-sideband modulator. The result is two sets of optical signals in separate fiber channels—the first containing light at frequencies *f*_1_ and *f*_2_, and the second at *f*_1_ + Δ and *f*_2_, where Δ = 100 MHz is the acousto-optic frequency shift driven by a low-noise microwave source (Keysight E8257D).

Through the frequency discriminator approach, the two signals are partially de-correlated through the introduction of a time delay *τ*. When these time-delayed signals are mixed together, frequency fluctuations (and equivalently phase noise) are converted to a noise pedestal centered around Δ with a well-defined frequency-domain transfer function^68,69^. In this microwave-photonic implementation, this time delay is introduced via a kilometer-length fiber-optic delay line after one of the two sources. Each optical signal pair is converted to the microwave domain on a separate photodiode, and the two signals at *f*_1_ − *f*_2_ and *f*_1_ − *f*_2_ + Δ are mixed down to Δ using an amplifier (Sage SBP-7531142515-1010-E1) and W-band mixer (Millitech MB1-10). This signal is digitized using a fast radiofrequency spectrum analyzer (RFSA; Keysight N9030B). For further details on the measurement approach, see Supplementary Note 1.

The measured single-sideband phase noise ${\mathscr{L}}(f)={S}_{\phi }(f)/2$ of the heterodyne synthesizer is plotted in Fig. 2c, showing constant, low-noise performance from 75 to 100 GHz. Notably, the measured phase noise level of −109 dBc/Hz at 100 kHz offset compares favorably with existing electronic synthesizers in this frequency range (see Supplementary Note 5 for details), and represents breakthrough performance for direct heterodyne microwave generation from two lasers. This measurement is corroborated by direct spectral analysis of the source at 20 GHz (see Supplementary Note 1). At low offsets, the phase noise of this free-running source can be suppressed by locking to a reference oscillator^40^. Noise spikes above 1 MHz arise from to the use of low-power switching regulators in the laser control electronics that operate in this frequency range, and may be mitigated through alternative electrical design.

To investigate the behavior of the millimeter-wave source in greater detail, we performed separate phase noise measurements of the two lasers using a self-heterodyne optical frequency discriminator experiment (see Supplementary Note 2 for details). The measured phase noise level of each laser is plotted as the red and violet lines in Fig. 2d; as expected from the down-mixing operation, their sum (black) matches precisely with the measured microwave phase noise at offsets above 1.5 kHz. Below frequency offsets of 1.5 kHz (shaded gray), excess fiber phase noise from environmental vibrations prevents accurate noise measurements. Throughout these measurements, one laser exhibits about 4 dB lower frequency noise than the other, implying that fundamental performance limits have not been reached^65^. As a result, improved laser noise, and hence synthesizer noise level, should be possible with fabrication process improvements.

Finally, we characterized the Allan deviation of the free-running heterodyne synthesizer, as plotted in Fig. 2e as a function of observation time. This measurement is performed through a combination of direct frequency counter measurements (for times above 0.1 s) and through calculations from the phase noise of Fig. 1c (for times below 0.1 s) normalized to a center frequency of 100 GHz. The frequency instability of the heterodyne source is directly set by laser frequency drifts (around a center frequency of 193.4 THz), and may be improved through locking to a stable external frequency reference if necessary.

### Field testing in a continuous-wave Doppler radar

Thus far, we have demonstrated microwave and millimeter-wave generation up to 104 GHz via optical heterodyning, and directly characterized the low-noise performance of our source in the microwave W band through laboratory measurements. Next, we experimentally show how this simple and high-performance microwave-photonic synthesizer can improve the dynamic range of a W band Doppler radar through outdoor testing.

Radars operating in the millimeter-wave W (75–110 GHz) and G bands (110–300 GHz) are useful for numerous applications including automotive sensors^70^, high-resolution ranging^23^, and remote cloud and precipitation sensing^17,18,71–73^. In terms of Earth science, satellite-based W-band radar instruments, such as CloudSat^71^ and the Doppler-capable Cloud Profiling Radar under development as part of EarthCARE^74,75^, provide data on atmospheric thermodynamics to elucidate how clouds and aerosols contribute to climate processes. Looking forward to future radar instrument concepts, access to spectral absorption features above 100 GHz will permit differential-absorption profiling of water vapor within clouds and precipitation, providing high-resolution data to enhance climate and weather modeling^18,73,76^. However, the potential performance of such millimeter-wave radars is highly sensitive to source purity. Cascaded frequency multiplication steps conventionally used to generate millimeter-wave signals greatly increase signal phase noise, which directly impacts the achievable measurement dynamic range in pulse-modulated radars. As a result, new low-noise millimeter-wave sources are necessary to enhance capabilities in future radar instruments.

**Fig. 3:**
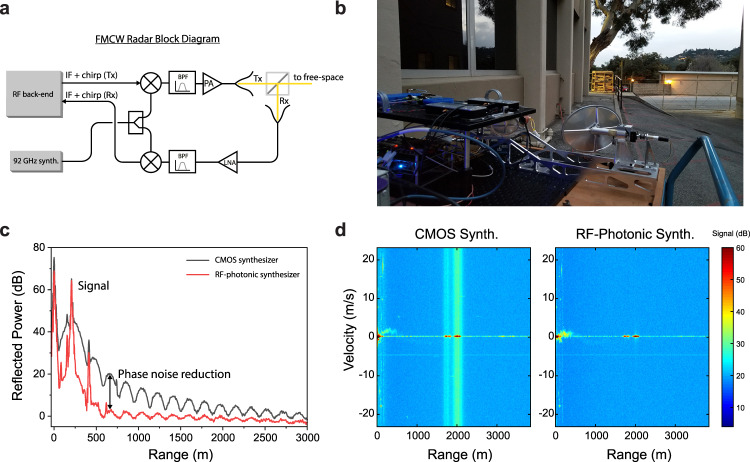
Field-testing of the heterodyne synthesizer in a 95-GHz continuous-wave radar. **a** Simplified block diagram of the frequency-modulated continuous-wave (FMCW) radar. A millimeter-wave source at 92 GHz is mixed with a frequency-chirped modulation waveform around an IF of ~3 GHz. A band-pass filter (BPF) rejects un-wanted mixing components, and the signal is amplified through a W-band power amplifier (PA) before being sent to the transmitter optics. Back-reflected signals are demultiplexed through polarization duplexing and passed through a low-noise amplifier (LNA) and BPF, and are mixed with the 92 GHz source down to the IF, prior to de-modulation and digitization in the back-end electronics. **b** shows a photograph of the radar test-bench with the primary aperture pointed at a distant hill, with the heterodyne synthesizer breadboard mounted on top. **c** plots measured back-reflection data from a building ~207 m away, when using either the radar’s existing 92 GHz CMOS synthesizer (dark gray) or the microwave-photonic heterodyne synthesizer (red). Phase noise fringes are significantly suppressed when using the low-noise photonics-based source. **d** plots Doppler data when the radar is pointed at a distant hill as in (**b**). Weak signals from drizzle are visible as slow-moving signatures at ranges closer than 1 km. When using the relatively noisy CMOS synthesizer (left), bright reflections from stationary hills lead to strong Doppler artifacts that would obscure the observation of co-located moving objects. By contrast, when using the heterodyne synthesizer (right), these artifacts are practically eliminated.

To demonstrate the utility of the heterodyne synthesizer for millimeter-wave remote sensing, we experimentally characterized its performance through field tests in a W-band radar. For these outdoor measurements, we used JPL’s 95 GHz Doppler radar that is part of its Gas and Ice Spectrometer/Radar (GAISR) instrument^77^. GAISR was originally designed as a prototype tool for probing cometary plumes, but is capable of detecting clouds, precipitation, and other terrestrial targets. Notably, its FMCW radar utilizes all-solid-state technology to achieve high sensitivity while minimizing system mass (6 kg excluding control computer) and power consumption (~22 W) for payload/platform-constrained applications. The basic operation scheme of GAISR’s W-band radar is depicted in the block diagram Fig. 3a. A 95-GHz, frequency-modulated waveform is synthesized by mixing a local oscillator (LO) tone directly generated at 92 GHz with a chirped waveform around an intermediate frequency (IF) of 3 GHz. This signal is amplified to around 0.6 W and transmitted through a 15 cm diameter antenna. Both transmit and receive horns share the same antenna through a form of quasioptical polarization duplexing^77^. In this configuration, the transmitted waveform is right-hand circular polarized by a grating reflector. Back-reflections from isotropic targets are converted to left-hand circular polarization, which are de-multiplexed using a wire grid that acts as a polarizing beam-splitter. This approach simplifies system design and alignment while providing >80 dB of T/R isolation necessary for high-sensitivity operation. (For full details, see ref. ^77^). Received echo signals are down-converted to the 3 GHz IF using the same 92 GHz LO as in the transmit chain. Following further down-conversion to base-band, a digital subsystem performs FMCW de-ramping and Doppler processing to generate range- or range-Doppler maps of targets. With an FMCW chirp bandwidth of 20 MHz and coherent processing of 512 consecutive 0.034 ms chirps, GAISR achieves range and velocity resolutions of 7.5 m and 0.1 m/s, respectively (see ref. ^77^ for details).

Field tests were carried out in a parking lot outside our laboratory building on March 9, 2020, as shown in Fig. 3b. For the 92 GHz local oscillator, we either used: (1) the GAISR instrument’s compact and low-power but relatively high-phase-noise silicon complementary metal-oxide-semiconductor (CMOS) electronic synthesizer, or (2) the photonic heterodyne synthesizer mounted on an optical breadboard. Figure 3c compares received signals when using each of the two W-band sources with the radar detecting a bright reflection from a building at a distance of 207 m. When using the silicon CMOS synthesizer, the building signal carries with it very strong range sidelobes that originate from the radar’s 92 GHz LO phase noise interfering with itself at the W-band down-converting mixer. (This interference process is analogous to the frequency discriminator measurement; for full details see Supplementary Note 3.) These strong artifacts hinder the radar’s performance by limiting its dynamic range close to bright targets. In this case, potentially weakly scattering objects between the radar and the building will be masked by the phase-noise-induced range sidelobes. By contrast, when using the heterodyne synthesizer, the sidelobes are strongly suppressed across the range axis; the level of suppression is related to the phase noise reduction vs. offset frequency, and reaches levels greater than 20 dB (100 × ) close to the target. Further details on the ranging measurement and related phase noise are discussed in Supplementary Note 3. In fact, the remaining low-level noise lobes are consistent with the phase noise from the intermediate-frequency 3 GHz oscillator used in the radar’s chirp synthesis (Abracon ASGTX). Replacing this with a lower-noise electronic source, such as a phase-locked dielectric resonator oscillator, would further reduce these phase noise lobes to approach the performance limit of the microwave-photonic heterodyne synthesizer.

As a second example of how the microwave-photonic synthesizer improves GAISR’s performance, we operated the radar in its range-Doppler mode while pointed at a hillside approximately 2 km away as in Fig. 3b. As plotted in Fig. 3d, strong reflections are detected from the hillside on the zero-velocity axis, while light drizzle is visible at ranges up to ~1 km with Doppler velocities <2 m/s. When using the relatively noisy CMOS synthesizer source (left), the zero-velocity hillside signals carry with them strong phase noise artifacts that spread across all measured velocities. This effect would make it difficult to detect weakly scattering moving targets close to the hillside, such as automobiles or pedestrians. By contrast, when using the low-phase-noise heterodyne source (right) the band of noise across the velocity span is practically eliminated. This results in significantly enhanced dynamic range for Doppler measurements in the presence of bright scatterers. Note that, due to the time delay between the two measurements, the drizzle signal is different between the two measurements; during the latter measurement with the heterodyne synthesizer, it is moving with a lower velocity relative to the stationary radar. In both plots, a constant-velocity spur is visible around −5 m/s; this possibly arises from the radar’s digital chirp synthesis.

The performance benefit enabled by the heterodyne synthesizer results from its ability to directly operate at high carrier frequencies with low phase noise. This contrasts with conventional techniques for W-band frequency synthesis based on frequency-multiplied electronic oscillators, which experience increases in phase noise level ∝ *M*^2^, where *M* is the multiplication factor. Thus, when looking to higher-frequency systems such as those operating at G-band or THz frequencies^72,73^, photonics-based signal generation permits even greater relative benefits. During our W-band radar comparisons, it is important to note that GAISR’s silicon CMOS synthesizer was selected for its ability to directly synthesize W-band signals while consuming less than 2 W of DC power; however, it does not offer optimized phase noise performance. While much lower phase noise electronic synthesizers are available, they do not typically support such power-efficient W-band signal generation. So, while the heterodyne synthesizer exhibits far lower phase noise than GAISR’s low-power CMOS W-band synthesizer, its noise level more importantly compares favorably to that of commercial low-noise electronic synthesizers frequency-multiplied to W-band (see Supplementary Note 5). Since most of the photonic heterodyne synthesizer’s modest (<6 W) power budget is used for thermal control, we anticipate that improved thermal management enabled by future miniaturization should allow it to operate at comparable powers to GAISR’s CMOS synthesizer while maintaining the same phase-noise benefit.

## Discussion

We have demonstrated a heterodyne microwave synthesizer based on down-mixing of two ultralow-noise, self-injection-locked lasers, and used this source to improve the performance of a millimeter-wave Doppler radar. Using a commercial fast photodiode, this source supports >100 μW output power from 1 to 104 GHz, and low-phase-noise operation (−109 dBc/Hz at 100 kHz offset; −129 dBc/Hz at 1 MHz offset) across this operating frequency range. These phase noise levels are comparable to those of high-end electronic synthesizers (e.g., Keysight E8257D, Micro Lambda Wireless MLVS-0520) multiplied to W-band. Additional details on the phase noise level of this synthesizer as it compares to a number of electronic and photonic sources are discussed in Supplementary Note 5. To further improve the close-in phase-noise of the free-running heterodyne synthesizer, frequency-locking to a stable reference can be implemented^40^.

Because the phase noise level of the heterodyne source is independent of operating frequency, this same performance can be scaled to sub-millimeter-wave or THz frequencies by replacing the commercial photodiode with a high-efficiency THz photomixer^78–80^. Prior implementations of heterodyne synthesis for THz waves^60^ have been limited by the noise performance of off-the-shelf lasers to use cases where suitable electronic oscillators are not available. By contrast, the ultralow noise performance enabled by self-injection-locked lasers opens the door to widely tunable heterodyne synthesizers with performance far superior to electronic sub-millimeter-wave sources.

While this first-generation demonstration was implemented on an optical breadboard, the system and component architecture is amenable to device-scale miniaturization based on microscale optics. Integrated packaging may also provide further improvements in noise performance, and reduce the power budget for thermal tuning. Currently, this source (excepting the external power supply) can be packaged in a form factor of 8 × 3 × 2 in^3^, with a straightforward path in the future for in^3^-level component miniaturization^51^; such a synthesizer is currently under development with the prototype design pictured in Fig. 1e.

For radar measurements of scenes with a large dynamic range of reflectively, it is important that the microwave source have very low phase noise to suppress noise artifacts from bright background signals, particularly when implementing pulse-compression techniques such as frequency modulation^81^. This situation occurs for satellite or airborne measurements of low-altitude clouds in the planetary boundary layer, where radar reflection from the earth’s surface can be many orders of magnitude brighter that clouds^18,77^. As demonstrated through our field testing, the heterodyne synthesizer, which generates low phase noise signals directly at high carrier frequencies, opens up a new technology space for these types of systems. As a result, it may enable higher resolution and sensitivity for future air- and spaceborne atmospheric radars operating at high frequency bands^72,73^. A number of civilian and national security applications can also benefit from the same technology enhancement^11,70,82,83^.

More generally, photonics offers numerous potential benefits for future radar systems in terms of broadband operation, performance, and re-configurability. Building on these capabilities, recent demonstrations of microwave photonic radars have implemented frequency agility^24,84^, ultrawide signal bandwidth^23^, and advanced signal processing^27,85^. So far, photonics-based radars have used either bulky and complex mode-locked lasers or external microwave sources for local oscillator synthesis. The heterodyne source demonstrated here represents an alternative approach, completely eliminating the need for high-frequency electronic synthesizers or external optical modulators and enabling greatly reduced system complexity and size for future compact microwave-photonic radars. Frequency-agile signal generation is achieved without the use of tunable optical filters or modulators as in many other photonic synthesizer schemes^24,84^. Additionally, the direct frequency modulation capability of the self-injection locked lasers can be used to implement chirp synthesis in the photonic domain (see Supplementary Note 4). Altogether, this synthesizer represents a potential enabling technology for photonics-based radars that require performance metrics beyond what is possible with all-electronic means, while also utilizing a simple and agile approach to frequency synthesis that allows it to operate as a drop-in replacement within existing compact millimeter-wave radar instruments. Looking forward, these results show how low-noise continuous-wave laser synthesizers can form the backbone of low-power and frequency-agile photonics-enabled radars.

In summary, we have demonstrated a low-noise, tunable heterodyne microwave and millimeter-wave synthesizer, and used this system to improve the performance of a FMCW radar in field tests. Because the RF signal is synthesized from 1.55 μm lasers, this system is intrinsically compatible with low-loss signal transmission and distribution over fiber, while reducing sensitivity to environmental noise and electromagnetic interference. Continuous tunability from 1 to 104 GHz in the same system may enhance heterodyne spectrometer technologies and frequency-agile millimeter-wave communications. Finally, by replacing the existing photodiode with a high-frequency THz photomixer, this same frequency-agility and low phase noise level can be extended to beyond 2 THz, outpacing the phase-noise performance of all-electronic sources in this range by many orders of magnitude, and enabling new applications in precision spectroscopy and sensing. This heterodyne synthesizer represents a remarkable combination of excellent phase noise performance, wide frequency tunability, compactness, low power consumption, and ruggedness, opening the door to future space-based and terrestrial millimeter-wave instruments with superb sensitivity, flexibility, and bandwidth.

## Supplementary information

Supplementary Information

## Data Availability

The data that support the plots within this paper and other findings of this study are available from the corresponding author upon reasonable request.
