# Supplementary material for: A low-noise photonic heterodyne synthesizer and its application to millimeter-wave radar
Source: Nat Commun. 2021 Jul 20;12:4397. doi: 10.1038/s41467-021-24637-0 (PMC8292534; doi:10.1038/s41467-021-24637-0)
Supplement: Supplementary file 1 — Supplementary Information [file 41467_2021_24637_MOESM1_ESM.pdf]

Supplementary Information: A Low-noise Photonic Heterodyne Synthesizer and its Application to Millimeter-wave Radar

Eric A. Kittlaus,[1] Danny Eliyahu,[2] Setareh Ganji,[2] Skip Williams,[2]
Andrey B. Matsko,[1] Ken B. Cooper,[1] and Siamak Forouhar[1]

[1] *Jet Propulsion Laboratory, California Institute of Technology, Pasadena, CA 91109 USA.*

[2] *OEwaves Inc., Pasadena, CA 91107 USA.*

SUPPLEMENTARY NOTE 1: MICROWAVE PHASE NOISE MEASUREMENTS

To characterize the RF phase noise of our heterodyne synthesizer, we implemented a microwave-photonic frequency discriminator measurement [1–4]. This approach is chosen because it is sensitive yet relatively simple, does not require an external low-noise reference oscillator, and is insensitive to frequency drifts in the free-running source. Because the signals are combined in optical fiber prior to heterodyning, it is also straightforward to implement a low-loss delay line, necessary for the frequency discriminator approach, in this system.

The phase noise measurement is diagrammed in Supplementary Fig. 1a. The two self-injection-locked lasers are tuned to frequencies f_1 and f_2, respectively, and are each split into two paths using fiber-optic directional couplers. One set of optical signals is immediately combined so that the resulting path guides optical tones at f_1 and f_2, identical to the normal configuration for heterodyne microwave synthesis. A frequency shift is implemented in one of the remaining paths using an acousto-optic frequency shifter (AOFS), resulting in an optical tone at $f_1 + \Delta$ that is then combined with the remaining tone at f_2. At this point, we have created two sets of optical signals in separate fiber channels; one channel carries light at frequencies f_1 and f_2, and the second at $f_1 + \Delta$ and f_2. A time delay τ is implemented in the first channel through the introduction of a fiber-optic delay line of length L (so $\tau = L/v_\mathrm{g}$, where v_g is the optical group velocity).

Next, each signal pair is converted to the microwave domain via optical heterodyning using fast photodiodes (Finisar XPDV4120R). The result is two microwave signals, synthesized from the same lasers, that are frequency detuned by Δ and time-delayed by τ. While the experimental setup to generate these signals is somewhat complex, it allows both the frequency-shift and the time-delay to be implemented in the optical domain with very low loss, noise, and spurious tones. Thereafter, these signals are mixed down to the offset frequency Δ using a W-band mixer (Millitech MB1-10). An additional power amplifier (Sage SBP-7531142515-1010-E1) is used to achieve the necessary local oscillator power for the mixer specification. Throughout these experiments, $\Delta = 100$ MHz and is generated by a low-noise microwave source (Keysight E8257D), with phase noise at least 15 dB less than the W-band heterodyne signal across the measurement range.

Through the frequency discriminator measurement, frequency fluctuations are converted into an output self-heterodyne noise signal with spectral content directly related to the source phase noise S_ϕ as [1, 3, 4]

$$S_\phi(f_\mathrm{m}) \ \ [\mathrm{dBr/Hz}] = S_\mathrm{d}(f_\mathrm{m}) - 20\log_{10}\left(\sqrt{2}\sin(\pi f_\mathrm{m}\tau)\right) - \mathrm{NBW}. \tag{1}$$

Here $S_\mathrm{d}(f_\mathrm{m})$ is the normalized self-heterodyne signal resulting from the frequency discriminator as a function of offset frequency f_m, and NBW is the noise bandwidth correction relative to the measurement resolution bandwidth of 1 Hz, equal to 0.24 dB for our spectrum analyzer (Keysight N9030B). In terms of single-sideband phase noise $\mathscr{L} \equiv S_\phi/2$, this expression can be written as

$$\mathscr{L}(f_\mathrm{m}) \ \ [\mathrm{dBc/Hz}] = S_\mathrm{d}(f_\mathrm{m}) - 20\log_{10}\left(2\sin(\pi f_\mathrm{m}\tau)\right) - \mathrm{NBW}. \tag{2}$$

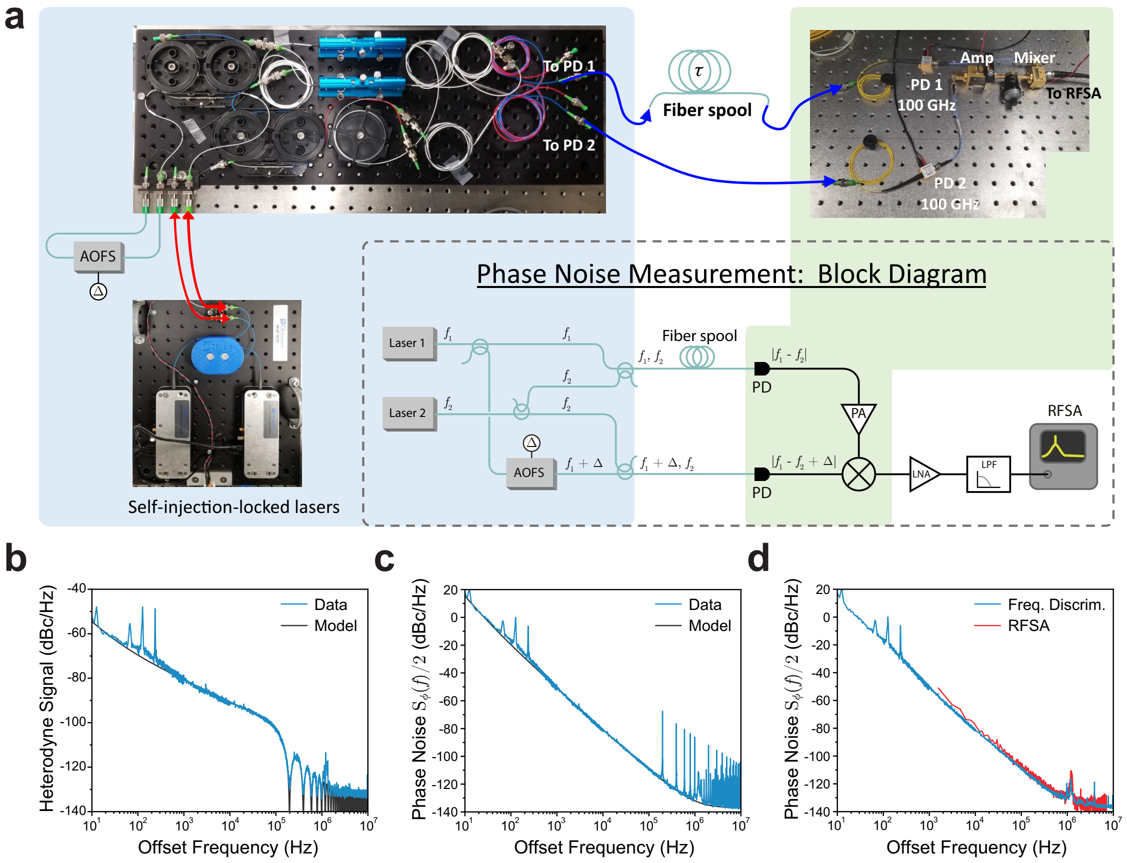

Supplementary Figure 1: Millimeter-wave phase noise characterization using a frequency discriminator measurement. (a) depicts the block diagram and benchtop layout of the phase noise measurement. Signal routing (light blue): Two frequency-detuned lasers are each split into two paths, and combined to form two copies of the same signal used for heterodyning, except that one optical tone is frequency-shifted by $\Delta = 100$ MHz using an acousto-optic frequency shifter (AOFS). One path is passed through a fiber-optic delay line to implement a time delay τ. Mixing (light green): The two frequency-detuned and time-delayed signals are mixed down to the microwave W-band using two fast photodiodes. These two microwave signals are then mixed down to the offset frequency Δ using a power amplifier and mixer. The output signal is amplified using a low-noise amplifier, filtered through a low-pass filter (LPF) and digitized using a radiofrequency signal/spectrum analyzer (RFSA). (b) plots the raw data (teal) as measured on the spectrum analyzer for a frequency $f_{\mathrm{RF}} = 100$ GHz and time delay $\tau = 5$ μs, showing a characteristic oscillatory spectral shape, and corresponding model (black). (c) plots these data after subtracting off the transfer spectrum. Sharp spurious peaks appear at multiples of $1/\tau$ corresponding to nulls of the frequency discriminator sensitivity. (d) plots the measured phase noise (teal) after merging with data sets with $\tau = 0.5$ μs and $\tau = 71$ ns to eliminate the spurious peaks at high offset frequencies. For comparison, data measured directly on a RFSA for a microwave frequency of $f_{\mathrm{RF}} = 20$ GHz are plotted in red, showing reasonable agreement. However, the direct spectrum analyzer measurement is challenging at low offset frequencies due to the frequency drift of the free-running source.

In this example, we perform measurements with the frequency of our heterodyne synthesizer tuned to $f_{\mathrm{RF}} = 100$ GHz. The output self-heterodyne signal for one such measurement with $\tau = 5.06$ μs is plotted in Supplementary Fig. 1b, and the corresponding phase noise data according to Eq. 2 is plotted in Supplementary Fig. 1c. For comparison, the black lines in Supplementary Fig. 1b-c show a fit corresponding to a simple

algebraic phase noise model:

$$S_\phi(f_\mathrm{m}) = k_0 + \frac{k_2}{f_\mathrm{m}^2} + \frac{k_3}{f_\mathrm{m}^3} + \frac{k_4}{f_\mathrm{m}^4} = 4.2\cdot 10^{-14}\,\mathrm{Hz}^{-1} + \frac{0.07\,\mathrm{Hz}}{f_\mathrm{m}^2} + \frac{1.7\cdot 10^4\,\mathrm{Hz}^2}{f_\mathrm{m}^3} + \frac{5.6\cdot 10^5\,\mathrm{Hz}^3}{f_\mathrm{m}^4}, \quad (3)$$

where k_0 represents the white phase noise floor, and k_2, k_3, and k_4 correspond to the strength of white frequency noise, flicker frequency noise, and random walk frequency noise, respectively. This analysis reveals that the phase noise behavior of this source is dominated by the k_3 (frequency flicker) noise component. At offset frequencies f_m that are multiples of $1/\tau$, the frequency discriminator experiences nulls in sensitivity, corresponding to sharp spurious peaks in the phase noise measurement of Supplementary Fig. 1c. To perform an accurate measurement over the entire frequency range, several delay lines with smaller values of τ are also used. Shorter delay lines have reduced sensitivity at small offset frequencies, but allow accurate measurement of the phase noise level at intermediate frequencies. For measurements at frequency offsets <100 kHz, a 1033 m fiber delay line is used (τ = 5.06 μs); for frequency offsets from 0.1 − 1 MHz, this is replaced with a 104-m-long fiber delay line (τ = 510 ns), and for frequency offsets greater than 1 MHz, a 14.5 m delay line is used (τ = 71 ns). Together, these lead to the data plotted in teal in Supplementary Fig. 1d, representing a phase-noise measurement from $f_\mathrm{m} = 10^1 - 10^7$ Hz.

For comparison, we tuned the frequency of the heterodyne synthesizer to f_RF = 20 GHz by thermally tuning one of the source lasers, and performed direct spectrum analyzer measurements of the phase noise. Performing such an accurate measurement is somewhat difficult due to the frequency instability of the free running source; however, the resulting data, plotted in red in Supplementary Fig. 1d, agree reasonably well with the results of the frequency discriminator experiment. Note that the phase noise produced through the heterodyne approach is frequency-independent because the output signal is generated through a down-mixing process.

Finally, it is worth noting that the characteristic oscillatory phase-noise interference signature of Supplementary Fig. 1b is analogous to the unwanted sidelobes observed during outdoor radar measurements in the main text. This is understood from the fact that these range sidelobes originate from the back-reflected (and time-delayed) W-band signal interfering with the local oscillator during down-conversion, corresponding directly to the time delay implemented in the fiber-optic frequency discriminator experiment. The magnitude and shape of these sidelobes is similarly determined by the system phase-noise level and target distance.

SUPPLEMENTARY NOTE 2: LASER PHASE NOISE MEASUREMENT

To separately characterize the optical phase noise of the self-injection-locked lasers, as in Fig. 2d of the main article, we construct a sub-coherence-length-delayed self-heterodyne interferometer that acts as an optical frequency discriminator [4]. In contrast to a traditional delayed self-heterodyne interferometer approach for characterizing laser linewidths, the sub-coherence optical frequency discriminator does not require an optical delay line that is longer than the laser coherence length, which becomes physically infeasible in the context

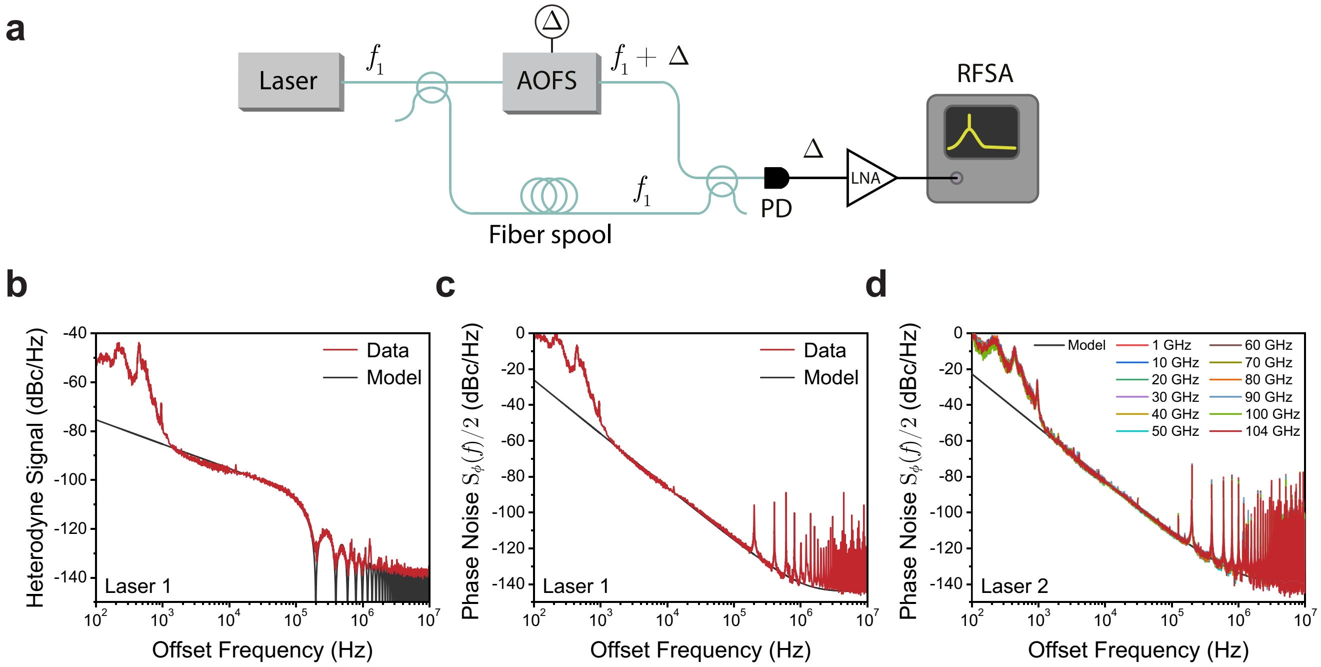

Supplementary Figure 2: Optical phase noise measurements based on a self-heterodyne frequency discriminator. (a) depicts the experimental apparatus. The laser under test at frequency f_1 is split into two paths. In one path, a frequency-shift to $f_1+\Delta$ is implemented using an acousto-optic frequency shifter. In the other, a time-delay is implemented through a long fiber-optic spool. The two signals are then incident on a fast photodiode mixing them down to the heterodyne frequency Δ and analyzed in the microwave domain using a radiofrequency spectrum analyzer. (b) plots the normalized self-heterodyne signal for one self-injection-locked laser ("Laser 1"; red) and a fit according to the model of Eq. 4 (dark gray). (c) plots the corresponding single-sideband phase noise data (red) and model (dark gray). Not that spikes above offset frequencies of 10^5 Hz correspond to nulls in the frequency discriminator sensitivity for a given delay time τ (d) Plots the measured single-sideband phase noise data for the second laser ("Laser 2") as its frequency detuning from Laser 1 is stepped from 1-104 GHz. Constant phase noise is observed across this tuning range, indicating that the phase noise of the synthesizer's RF output should also be constant as the output frequency is changed.

of Hz-linewidth lasers, and allows measurement of the full frequency/phase noise spectrum [3–5].

The operation scheme of this laser frequency noise measurement system, diagrammed in Supplementary Fig. 2a, is analogous to the microwave frequency discriminator of the previous section: frequency fluctuations (phase noise) in the source are converted to amplitude noise with a transfer function defined by the temporal delay. In practice, compared to the microwave measurement, the optical setup is much less complex: First, a laser at frequency f_1 is split into two paths. In the upper path, the laser is frequency-shifted by $+\Delta$ using an acousto-optic frequency-shifter driven by the same low-noise microwave source as before (Keysight E8257D) at $\Delta = 100$ MHz. In the lower path, the un-shifted laser tone is passed through a long fiber spool to implement a time delay. The two signals are then combined and incident on a fast photodiode, which acts as the mixer to down-convert the optical beat-note to Δ. The signal is amplified using a low-noise amplifier (LNA), and detected on a radiofrequency spectrum analyzer. In analogy to the microwave frequency discriminator, this setup creates two frequency-detuned copies of the same signal, implements a time delay between the two,

and mixes them down to a heterodyne frequnecy Δ, resulting in the same characteristic phase noise transfer function as in Eq. 2. However, this setup experiences a practical limitation, which is that it is highly sensitive to variations in the fiber path length, e.g. due to environmental noise. This can be understood by the fact that phase modulation in either interferometer arm is indistinguishable from additional laser phase noise [6]. By contrast, in the microwave-photonic delay line of Supplementary Fig. 1a, fiber noise equally modifies the phase of both optical tones carried over the fiber, and is hence common-mode-rejected during mixing on the photodiode.

Experimental results for one laser ("Laser 1" in Fig. 2d of the main article) are plotted in red in Supplementary Fig. 2b-c. The normalized self-heterodyne signal and corresponding single-sideband phase noise are plotted in red for a time delay $\tau = 5.06$ µs. Above frequency offsets $f_{\mathrm{m}} > 1$ kHz, the signal behavior is qualitatively similar to that of the heterodyne microwave source, as expected. However, below 1 kHz, strong fiber-induced phase noise obscures the intrinsic phase noise of the laser source. As before, sharp spikes corresponding to nulls in the frequency discriminator sensitivity are seen above 100 kHz, and can be eliminated by systematically varying the delay line length. These data can similarly be fit to a simple model

$$S_{\phi}^{\text{Laser 1}}(f_{\mathrm{m}}) = k_0 + \frac{k_2}{f_{\mathrm{m}}^2} + \frac{k_3}{f_{\mathrm{m}}^3} = 8 \cdot 10^{-15}\,\mathrm{Hz}^{-1} + \frac{0.012\,\mathrm{Hz}}{f_{\mathrm{m}}^2} + \frac{5.2 \cdot 10^3\,\mathrm{Hz}^2}{f_{\mathrm{m}}^3}. \tag{4}$$

Note that meaningful fitting of k_4 as in Eq. 3 is impossible due to the excess fiber noise. Practically speaking, the extremely small white frequency noise coefficient k_2 of this laser permits low-phase-noise performance at large frequency offsets. From $f_{\mathrm{m}} = 10^3 - 10^5$ Hz, the phase noise behavior of this source is dominated by the comparatively large k_3 frequency flicker coefficient. Similar fitting for Laser 2 (Supplementary Fig. 2d) reveals

$$S_{\phi}^{\text{Laser 2}}(f_{\mathrm{m}}) = 2.6 \cdot 10^{-14}\,\mathrm{Hz}^{-1} + \frac{0.052\,\mathrm{Hz}}{f_{\mathrm{m}}^2} + \frac{1.1 \cdot 10^4\,\mathrm{Hz}^2}{f_{\mathrm{m}}^3}. \tag{5}$$

As expected for the photomixing process, Eqs. 4-5 approximately sum to give the total microwave phase noise in Eq. 3. Note that the measured optical phase noise for Laser 2 in Supplementary Fig. 2d is constant as its frequency detuning relative to Laser 1 is varied over the synthesizer's tuning range; as a result, the phase noise level of the output RF signal that results from laser heterodyning should be independent of operation frequency.

A. Laser Linewidth and Phase Noise

The spectral bandwidth of laser oscillators is often specified in terms of a single full-width at half-maximum linewidth Δf_{FWHM}. While this can be an over-simplification for many laser sources with nontrivial noise spectra, the linewidth is well-defined for an ideal quantum-limited (Schawlow-Townes-like) oscillator. In this case, a laser exhibits white frequency noise with constant spectral density $S_{\Delta f} = k_2$. The corresponding phase noise spectral density as a function of offset frequency is $S_{\phi}(f_{\mathrm{m}}) = \frac{k_2}{f_{\mathrm{m}}^2}$, and the optical lineshape is

a well-defined Lorentzian with $\Delta f_{\mathrm{FWHM}} = \pi k_2$ [7]. This same definition for Lorentzian linewidth is used in different contexts to specify the "fundamental" or "instantaneous" linewidth of a laser source, referring to its minimum linewidth as set by white quantum-noise processes [5].

By contrast, flicker-dominated lasers such as those used in our heterodyne synthesizer do not have one strictly-defined linewidth. One convention [8] notes that in the white frequency noise case, the total root-mean-square phase fluctuation for all frequencies larger than the laser linewidth is $1/\pi$ rad^2. Therefore, one equivalent definition for the integral (as opposed to instantaneous) linewidth of a laser with an arbitrary phase noise spectrum is given by

$$\int_{\Delta f_{\mathrm{FWHM}}}^{\infty} S_\phi(f_m) df_m = 1/\pi. \tag{6}$$

Similar definitions choose different endpoints for the integral of the phase noise wing, but lead to similar results [9, 10]. Through this calculation, the linewidth of a frequency-flicker dominated laser is $\Delta f_{\mathrm{FWHM}} = \sqrt{\pi k_3/2}$. The corresponding integral linewidth of Laser 1 (Eq. 4) is 90 Hz. By contrast, considering only the white frequency noise component of this laser gives an instantaneous linewidth $\pi k_2 \approx 38$ mHz. Importantly, despite a relatively modest integral linewidth, the sharp rolloff $\propto f_{\mathrm{m}}^{-3}$ of this laser's phase noise and its extremely low white frequency noise coefficient k_2 result in very low noise levels at frequency offsets > 10 kHz as compared to conventional lasers. This narrow (sub-Hz) instantaneous linewidth is particularly amenable to our application of FMCW radar, as well as other applications where short-term coherence is paramount such as coherent communications. Notably, Laser 1 exhibits a phase noise below that of an ideal 1-Hz Lorentzian linewidth laser above $f_m \approx 5$ kHz.

SUPPLEMENTARY NOTE 3: FMCW RADAR PHASE NOISE AND RANGING

In the main text, we showed through field tests that the low phase noise of the photonic heterodyne synthesizer can improve the measurement dynamic range of an FMCW radar. Here, we quantitatively investigate the impact of phase noise on the ranging measurement.

To understand the origin of phase-noise related ranging artifacts, it is helpful to briefly review the FMCW radar operation concept. GAISR's FMCW radar implements a 20 MHz frequency ramp over a repetition period of $T_{\mathrm{rep}} = 34$ μs on the transmitted signal, corresponding to a chirp rate $k = 0.588$ MHz/μs. During ranging measurements, received back-reflected (and hence time-delayed) echoes are digitally mixed with a reference chirp-modulated waveform with the same chirp rate. This down-mixing maps target distance d (time delay $\tau_{\mathrm{d}} = 2d/c$) to detected beat frequency $f_{\mathrm{d}} = k\tau_{\mathrm{d}} = 2dk/c$, which is around $3.92 \cdot d$ MHz/km for these measurement parameters. A fast Fourier transform (FFT) on each mixed waveform resolves targets in range. Next, these range-compressed spectra are stacked in order of the chirp pulse sequence, and a second FFT along this "slow time" dimension separates targets according to their Doppler shift, enabling GAISR's range-Doppler functionality.

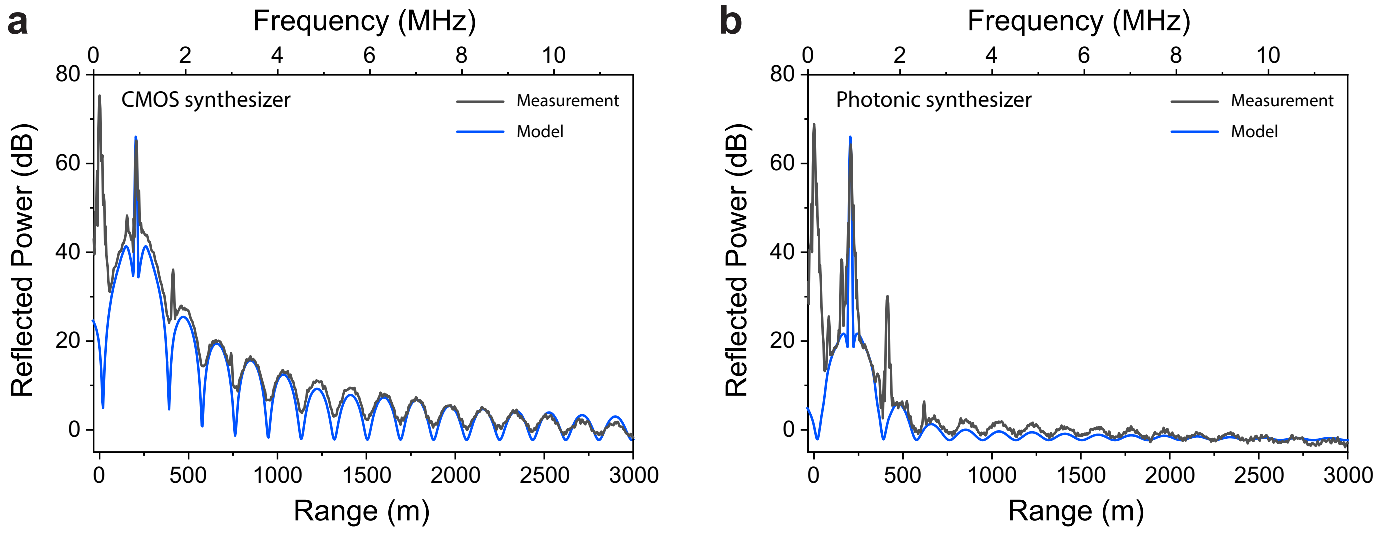

Supplementary Figure 3: Impact of phase noise on FMCW radar measurements. (a) plots the measured signal (gray) for a ∼207 m distant target obtained using with GAISR's FMCW radar while using the silicon CMOS synthesizer as the radar's W-band local oscillator. The predicted signal assuming the model of Eq. 7 is shown in blue, exhibiting strong ranging side-lobes arising from the source's phase noise. (b) plots the measured (grey) and predicted (blue) signal when using the photonic heterodyne synthesizer, displaying greatly reduced ranging artifacts. The calculated signal is based on the phase noise of GAISR's 3 GHz chirp synthesizer, which is around 20 dB greater than that of the heterodyne synthesizer operating at 92 GHz and is understood to set the minimum noise level of this radar measurement.

In practice, the detected signal from even an ideal target is not perfectly monochromatic due to frequency variations (phase noise) in the carrier signal over the delay period. When the signal is mixed with a time-delayed version of itself, this phase noise is converted to a spectral 'pedestal' extending across the frequency/range axis. This situation is exactly analogous to the lab-based frequency discriminator measurement used to quantify oscillator phase noise. In the context of a radar measurement, this phase noise signature limits the measurement dynamic range in the vicinity of bright targets or background signals [11, 12], such as ground reflections in a nadir-pointing radar. Carrier phase noise also impacts GAISR's range-Doppler mode, since uncorrelated phase-jitter over the inter-pulse repetition period manifests as white noise stretching along the velocity axis. Generally speaking, accurate measurement of scenes containing a range of object reflectivities motivates the need for high-frequency synthesizers with low phase noise at frequency offsets > 10 kHz.

From Eq. 2, we can calculate the power spectrum (in relative units of dBr/Hz) of the noise signal $S_d(f)$ accompanying an ideal point target,

$$S_d(f) = \mathscr{L}\left(|f - f_0|\right) + 10\log_{10}\left(4\sin^2(\pi\tau\left(f - f_0\right))\right) + 10\log_{10}\left(\Delta f\right). \tag{7}$$

Here f is the demodulated frequency, f_0 and τ are the center frequency and round-trip time delay of the signal, and $\Delta f = 1/T_{\rm rep}$ is the radar's effective detection bandwidth. This analysis shows that bright reflections produce an oscillatory noise signature along the frequency (range) axis with a power spectral density

proportional to the phase noise $\mathscr{L}$ of the radar's transmitted signal.

We experimentally verify this model by comparing it to the ranging data from the main text. In those measurements, GAISR's FMCW radar is pointed at a building around 207 m away, and the back-reflected signal is detected, as shown in Supplementary Fig. 3. The demodulated signals resulting from two different source synthesizers are compared to that calculated from Eq. 7 assuming a fixed instrument noise floor and measurement bandwidth $\Delta f = 29.4$ kHz. For the measurement made with GAISR's W-band silicon CMOS synthesizer (gray curve in Supplementary Fig. 3a), this synthesizer's phase noise is used to calculate the corresponding expected signal (blue). (This synthesizer has a phase noise level around -68 dBc/Hz at 100 kHz offset and -91 dBc/Hz at 1 MHz.) Good agreement is observed.

For the measurement made using the photonic heterodyne synthesizer (gray in Supplementary Fig. 3b), we calculate the expected signal (blue) using the phase noise of the radar's auxiliary 3 GHz oscillator used to offset the signal from baseband during chirp synthesis. This crystal oscillator (Abracon ASGTX) has phase noise around 10-20 dB higher than that of the photonic synthesizer across the measured frequency range. For example, the ASGTX's output signal at 3 GHz has a phase noise level around -87 dBc/Hz at 100 kHz offset and -111 dBc/Hz at 1 MHz, which is then mixed on to the 92 GHz signal from the heterodyne synthesizer. This phase noise level reduces receiver noise by around 20 dB at 25 m (100 kHz offset in demodulated frequency) and 250 m (1 MHz) away from the target relative to the silicon CMOS synthesizer in Supplementary Fig. 3a. A further ~20 dB improvement should be possible by using a lower-noise 3 GHz electronic oscillator to implement the chirp offset, or by synthesizing the frequency chirp in the photonic domain, so that the phase noise of the heterodyne synthesizer is the ultimate limiting factor.

SUPPLEMENTARY NOTE 4: FREQUENCY MODULATION

Each of the two lasers used to construct the heterodyne synthesizer allows for external frequency control through slow thermal and fast strain tuning via an SMA interface, permitting operation as a direct voltage-controlled oscillator. Strain tuning is produced using a piezoelectric transducer (PZT) that is directly laminated on the whispering-gallery-mode resonator (WGMR) used for self-injection-locking [13]. By modifying the effective indices of the resonator modes through the photoelastic (elasto-optic) effect, efficient direct frequency modulation (FM) is achieved.

In this section, we investigate the frequency modulation response of these lasers, and apply this technique to the generation of arbitrary FM waveforms. The capability for direct voltage-controlled frequency modulation may be particularly useful for the generation of chirped radar waveforms without the need for an external electronic chirp synthesizer.

B. Characterization of Modulation Response

We begin by characterizing strain-based frequency tuning and modulation through direct measurements of the output spectrum of the heterodyne synthesizer tuned to a center frequency of $f_{\mathrm{RF}} = 10$ GHz, while applying an external drive signal to the PZT. We first applied a DC voltage to the PZT, and observed a frequency tuning sensitivity $S_{\mathrm{DC}} = 15$ MHz/V over a voltage range of ±5 V. This direct frequency tuning could be utilized for fine-tuning of the synthesizer output frequency, or to implement frequency locking to an external reference.

Next, we performed frequency-domain measurements by applying a sinusoidal drive signal with peak voltage V_{p} and frequency f_{ext}. Under this configuration, the PZT enables strong frequency modulation (FM) of the laser frequency, and hence of the output RF signal from the heterodyne synthesizer. In the spectral domain, FM is manifested as energy transfer to frequency-detuned sidebands at $f_{\mathrm{RF}} \pm n f_{\mathrm{ext}}$, $(n = 1, 2, 3, \ldots)$, with a power in the nth sideband given by

$$P\left(f_{\mathrm{RF}} \pm n f_{\mathrm{ext}}\right) = P_{\mathrm{tot}} J_n\left(\beta\right)^2. \tag{8}$$

Here, P_{tot} is the total RF power output from the heterodyne synthesizer, J_n are the Bessel functions of the first kind, and β is the modulation index; β is proportional to the peak drive voltage V_{p} as

$$\beta = \frac{S V_{\mathrm{p}}}{f_{\mathrm{ext}}}, \tag{9}$$

where S is the FM sensitivity in units of MHz/V.

We first applied a very weak drive $V_{\mathrm{p}} \approx 0.1$ mV, resulting in narrowband FM with appreciable power transfer only to the $n = \pm 1$ sidebands, allowing direct determination of β. The results of these measurements are plotted in Supplementary Fig. 4a, normalized relative to V_{p}. The corresponding calculated FM sensitivity S is plotted in Supplementary Fig. 4b. For frequencies $f_{\mathrm{ext}} = 10$-500 kHz, β follows the expected $1/f\mathrm{ext}$ trend (S is constant). Across this range, S agrees with the measured DC sensitivity $S_{\mathrm{DC}} = 15$ MHz/V. However, above $f_{\mathrm{ext}} = 1$ MHz, acoustic resonances of the WGMR modify the modulation strength. Notably, strong resonant enhancement of frequency modulation is observed around $f_{\mathrm{ext}} = 1214$ kHz, corresponding to a resonant elastic mode with a bandwidth of ~ 30 kHz.

Next, we applied a stronger drive signal to demonstrate wideband FM and comb generation, as shown through the spectrum analyzer traces in Supplementary Fig. 4c-f. Due to the large FM sensitivity through piezoelectric modulation, efficient frequency modulation is observed even at low drive voltages $V_{\mathrm{p}} < 1$ V. (Through FM, the resulting signal bandwidth is $\Delta f \approx 2 f_{\mathrm{ext}}\left(\beta + 1\right)$; βf_{ext} corresponds to the maximum instantaneous frequency deviation from the carrier.) At a modulation frequency $f_{\mathrm{ext}} = 200$ kHz, power transfer to around 60 (200) spectral lines is produced at a drive voltage of 0.4 V (1.25 V). Around the resonant frequency of $f_{\mathrm{ext}} = 1214$ kHz, around 250 (780) spectral lines are observed at a drive voltage of 0.4 V (1.25 V). The latter corresponds to an electrical frequency comb with a bandwidth approaching 1 GHz.

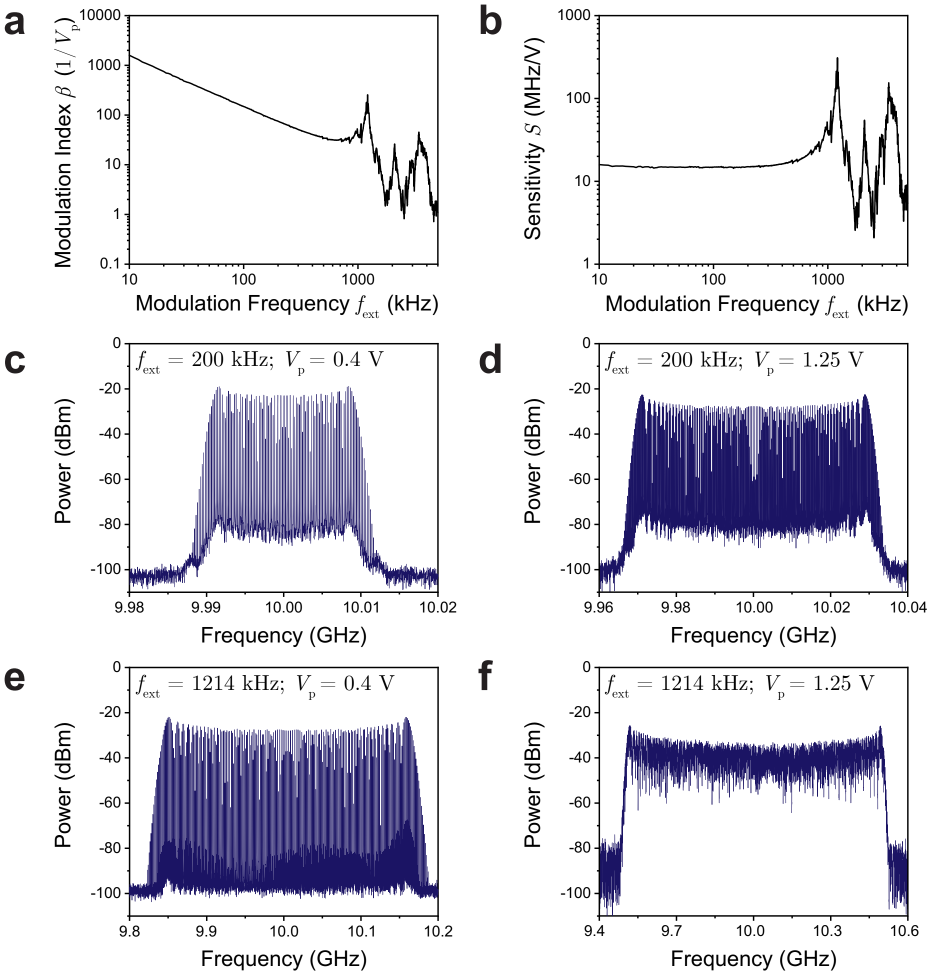

Supplementary Figure 4: Frequency modulation via piezoelectric tuning. (a) plots the measured modulation index β as a function of sinusoidal modulation frequency f_{ext}. Data are normalized relative to the peak modulation voltage V_{p}, and display the expected $1/f_{ext}$ trend for FM at low modulation frequencies. Above $f_{\text{ext}} = 1$ MHz, acoustic resonances of the WGMR modify the observed modulation strength. (b) shows the same data in terms of modulation sensitivity S, describing the peak frequency shift as a function of applied voltage. From 10-500 kHz, $S \approx 15$ MHz/V. (c) and (d) plot the measured RF spectra (resolution bandwidth = 5 kHz) when the heterodyne synthesizer is tuned to a center frequency of 10 GHz, and the PZT of one laser is driven at $f_{\text{ext}} = 200$ kHz with a peak voltage of $V_{\text{p}} = 0.4$ V (c) and $V_{\text{p}} = 1.25$ V (d). Individual spectral lines are clearly resolved. (e) and (f) plot the measured RF spectra when the PZT is driven at $f_{\text{ext}} = 1214$ kHz, corresponding to the strong acoustic resonance observed in (a-b), with a peak voltage of $V_{\text{p}} = 0.4$ V (e) and $V_{\text{p}} = 1.25$ V (f). In (e), the spectrum analyzer's resolution bandwidth is 5 kHz, while in (f) it is set to 510 kHz, and the individual spectral lines are not resolved.

C. Arbitrary Frequency-modulated Waveform Synthesis

Flexible frequency-modulated waveform synthesis is useful for pulse-compressed and continuous-wave radars, as well as a variety of optical and microwave communications and sensing applications. In radar systems, this is commonly achieved by mixing a high-frequency local oscillator with a low frequency chirped waveform from an auxiliary electronic synthesizer. Here, we investigate the ability of our synthesizer to di-

rectly generate arbitrary FM waveforms based on laser frequency modulation. This capability could be used to simplify the system design of a future photonics-based radar.

We begin by implementing a number of simple FM waveforms and characterizing the corresponding output modulated signals. In each case, the PZT is driven by a 10 kHz signal from an arbitrary waveform generator (AWG) with an amplitude of 1 V_{pp} (one Volt peak-to-peak). This produces a peak frequency deviation of around ±7.5 MHz over each 100 µs modulation (or 'pulse') period. Throughout these measurements, the synthesizer is tuned to a center frequency of $f_c = 20$ GHz to enable direct signal capture by our relatively low-frequency RF spectrum/signal analyzer (Keysight N9030B); however the same modulation response can be implemented at any frequency up to 104 GHz within the synthesizer's tuning bandwidth. We capture both the output RF spectrum, and the full down-mixed modulated waveform in signal analysis mode, with a sampling rate of 32 ns (31.25 MHz). The captured waveform can be represented in complex coordinates as

$$R(t)e^{i\phi(t)} = R(t)e^{i2\pi(f(t)-f_0)} \tag{10}$$

where $R(t)$ and $\phi(t)$ are the signal amplitude and phase, and $f_0 \approx f_c$ is the signal analyzer's local oscillator frequency. From the unwrapped phase, we can calculate the instantaneous (demodulated) frequency as

$$f(t) = f_0 + \frac{1}{2\pi}\frac{d\phi}{dt}. \tag{11}$$

For ideal frequency modulation, $f(t) - f_c \propto V(t)$, although in practice some distortion or nonlinearity is often introduced due to the frequency response of the voltage-controlled oscillator.

The output signal for four different 10 kHz FM waveforms (sinusoidal, triangle wave, parabolic, and trapezoidal) is plotted in Supplementary Fig. 5. In each case, the top panel Supplementary Fig. 5a-d.i shows the instantaneous frequency (relative to the carrier frequency f_c) of the demodulated waveform (dark blue) atop the shape of the input voltage (light blue). For all of these waveforms, the output demodulated signal closely mirrors the temporal behavior of the input modulation voltage. In particular, for the triangle ramp of Supplementary Fig. 5b, the output modulation is highly linear, with a coefficient of determination $r^2 = 0.99993$ for a linear fit to each piecewise element. Supplementary Figs. 5a-d.ii plot the measured RF spectra corresponding to each case, while ideal spectra from numerical calculations considering the input voltage waveform are plotted below in Supplementary Fig. 5a-d.iii. Good agreement is observed.

Through these tests, the drive voltage (1 V_{pp}) and hence peak modulation frequency (∼15 MHz) were kept small due to the limited sampling bandwidth of our signal analyzer. However, as previously shown, much higher drive voltages (at least to 5 V_{pp}) are possible, leading to the potential for ∼100 MHz FM bandwidths. Through separate experiments, we tested triangle wave FM up to a with a drive voltage of 2.5 V_{pp}, corresponding to a FM bandwidth of 75 MHz, and observed no nonlinearity with respect to voltage in the FM response.

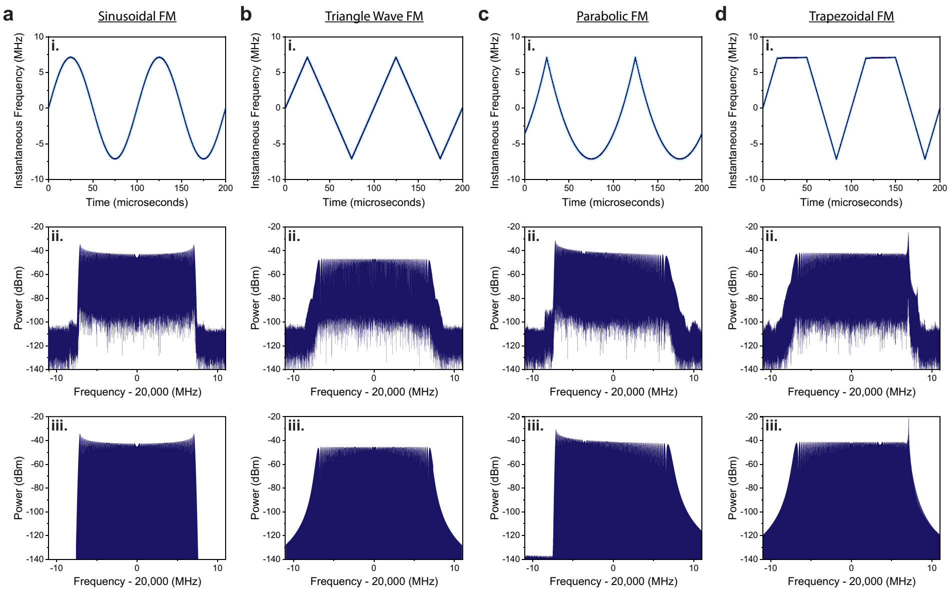

Supplementary Figure 5: Swept frequency modulation with various waveforms. Panels (a)-(d) plot data for sinusoidal FM (a), triangle wave FM (b), parabolic FM (c), and one form of trapezoidal FM (d). For each modulation type, subplot (i) depicts the instantaneous frequency deviation of the demodulated waveform in dark blue atop the waveform of the incident voltage (light blue) for comparison. Subplot (ii) shows the output spectrum around a center frequency of 20 GHz as measured on a RF spectrum analyzer with a resolution bandwidth (RBW) of 250 Hz, and subplot (iii) shows the expected spectrum calculated assuming ideal frequency modulation according to the input waveform.

D. Correction of FM Distortion via Pre-emphasis

So far, we have studied FM at relatively low frequencies (10 kHz) from continuous input voltage waveforms. However, higher-frequency modulation should be possible, as evidenced by the relatively-constant FM sensitivity up to around 500 kHz in Supplementary Fig. 4. To explore this possibility, we first investigate a 30 kHz triangle-wave modulation in detail, and carefully characterize the distortion introduced through the elasto-optic FM process. The input voltage is plotted in Supplementary Fig. 6a, resulting in the output demodulated signal in Supplementary Fig. 6b. Upon close inspection, very small oscillations in the demodulated frequency are revealed after each peak and trough of the triangle wave (linear fit $r^2 = 0.99984$). Such a non-linearity in the frequency ramp is undesirable since it would (for example) deteriorate the range resolution of a frequency-modulated radar.

To understand this distortion in more detail, one approach is to examine the Fourier decomposition of the demodulated waveform itself. Specifically, an ideal triangle wave of frequency f can be decomposed into a series of harmonics at odd multiples of f, with relative power amplitudes proportional to f^{-4}. However, the amplitudes (and phases; not pictured); of the Fourier components of the output demodulated signal deviate from this ideal situation. This can be readily seen in the dark blue curve of Supplementary Fig. 6c, which depicts the discrete Fourier transform of a sequence of captured data. Notably, the output frequency components around 1.2 MHz are dramatically enhanced compared to the ideal trend (red). This directly

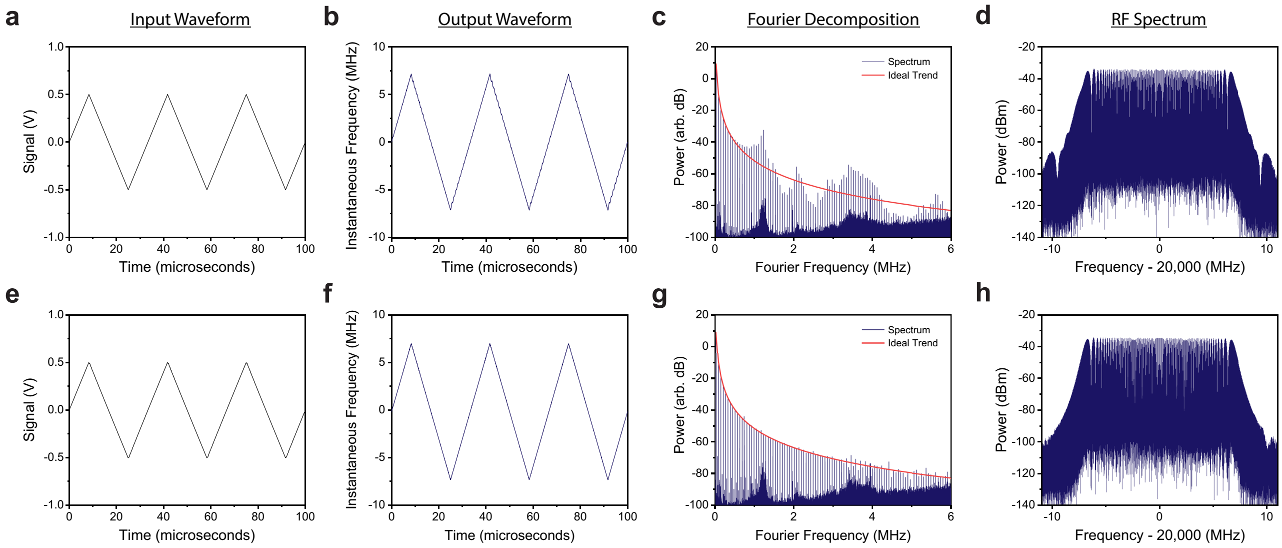

Supplementary Figure 6: Frequency modulation and response correction for a 30 kHz triangle wave. (a) Input voltage with amplitude of 1 V_{pp} (b) Resulting output demodulated signal, showing the triangle wave FM; however, some distortion is visible. (c) Fourier decomposition of the output waveform, showing the power amplitude of its constituent harmonics from 0-6 MHz (blue) and the expected trend for an ideal triangle wave (red). (d) The RF spectrum of the modulated wave around a carrier frequency of 20 GHz. (e) Input voltage corrected according to the method outlined in the text. (f) Demodulated signal, representing near-ideal triangle wave FM. (g) Fourier decomposition of this waveform; the measured power amplitude of the various harmonics (blue) closely matches the ideal trend (red). (h) RF spectrum of the corrected FM signal.

corresponds to the resonant feature observed in Supplementary Fig. 4a-b that arises from the WGMR's acoustic response.

A natural question to ask is whether this nonideal behavior can be corrected. We address this by postulating that the laser system's frequency modulation response follows a linear transformation relationship:

$$A_{\text{out}}\left(f\right) = \bar{S}\left(f\right) e^{i\Delta\phi(f)} A_{\text{in}}\left(f\right). \tag{12}$$

Here $A_{\text{out}}\left(f\right)$ and $A_{\text{in}}\left(f\right)$ represent the Fourier amplitudes of the output and input waveforms at frequencies f that are multiples of the fundamental frequency, $\bar{S}\left(f\right) = S\left(f\right)/S\left(0\right)$ is the normalized FM sensitivity, and $\Delta\phi\left(f\right)$ is a frequency-dependent phase rotation term. This ansatz allows us to directly calculate $S\left(f\right) e^{i\Delta\phi(f)}$ from the measured spectral content of the input voltage and output FM waveforms. Assuming that this transformation relationship is valid, it is then straightforward to calculate the input voltage wave that leads to a desired output waveform–by simply dividing each of its Fourier components by this frequency-dependent transfer function.

We use this 'pre-emphasis' approach to synthesize a corrected triangle wave as shown in Supplementary Fig. 6e-h. In this case, the calculated input voltage differs slightly from a triangle wave, and displays an additional notch (dip) after each peak (trough) that can be thought of as coherently cancelling the resonant acoustic response by driving out-of-phase relative to the acoustic ring-down. The resulting demodulated waveform

exhibits improved triangle-wave FM, as seen both by the excellent linearity displayed in Supplementary Fig. 6f (linear fit $r^2 > 0.99999$) and by the near-ideal Fourier amplitudes of the output waveform. For comparison, we also plot the RF spectra resulting from the uncorrected and corrected FM in Supplementary Fig. 6d and Supplementary Fig. 6h, respectively.

So far, we have considered frequency modulation waveforms that are continuous and relatively well-behaved. However, for many applications it is useful to consider discontinuous waveforms, such as the linear sawtooth ramp depicted in Supplementary Fig. 7. In addition to the triangle wave FM in the previous section, this sawtooth sweep is commonly employed in radar systems. Because this waveform experiences an abrupt frequency jump, its Fourier decomposition necessarily consists of many high-frequency components. When we attempt to implement frequency modulation using the input sawtooth voltage of Supplementary Fig. 7a, we find that the output demodulated signal is highly distorted, since this 30 kHz sawtooth ramp comprises numerous harmonic components well in excess of 1 MHz. This resonant distortion effect is strongly exacerbated by the fact that the Fourier amplitudes of these components fall off only as f^{-2}, plotted as the red line atop the calculated spectral decomposition of the demodulated waveform (dark blue) in Supplementary Fig. 7c. Strong aliasing is also visible from high-frequency spectral components beyond the signal analyzer's Nyquist frequency. The RF spectrum of the modulated signal (Supplementary Fig. 7d) is extremely distorted compared to that which would be expected from a simple periodic modulation.

We implement the same pre-emphasis approach to correct the output waveform, constructing an input voltage that is mapped to an output sawtooth FM by the laser's modulation response. Due to limited dynamic range in our measurement hardware, we perform two iterations according to Eq. 12 to accurately determine the values of $\bar{S}\,(f)\,e^{i\Delta\phi(f)}$. (In other words, we perform an initial attempt, and then correct according to the residual error in the output waveform.) The results are plotted in Supplementary Fig. 7e-h. The calculated input voltage in Supplementary Fig. 7e is programmed into the AWG, and produces the output demodulated signal in Supplementary Fig. 7f. This frequency ramp is significantly cleaner than the uncorrected output (Supplementary Fig. 7b), and its Fourier decomposition (dark blue) closely mirrors the ideal trend (red) in Supplementary Fig. 7g. The residual deviation from an ideal sawtooth FM is deterministic and stable over hour time-scales, indicating that further improvement should be possible.

Modulation correction via voltage pre-emphasis produces good results due to the excellent voltage-linearity and thermal stability of the elasto-optic FM mechanism. This form of distortion compensation is of particular interest for our target application of frequency-modulated radar since a single, highly-repeatable modulated waveform is typically employed. In our system, FM distortion arises from acoustic resonances within the WGMR structure. Alternately, this particular issue can be completely eliminated by switching to direct electro-optic modulation of a WGMR fabricated from a suitable electro-optic material [14]. This could also enable much wider bandwidth FM, since highly-linear electro-optic modulation can readily be achieved from DC-GHz frequencies. In either case, residual non-linearity of the frequency ramp may also be corrected on

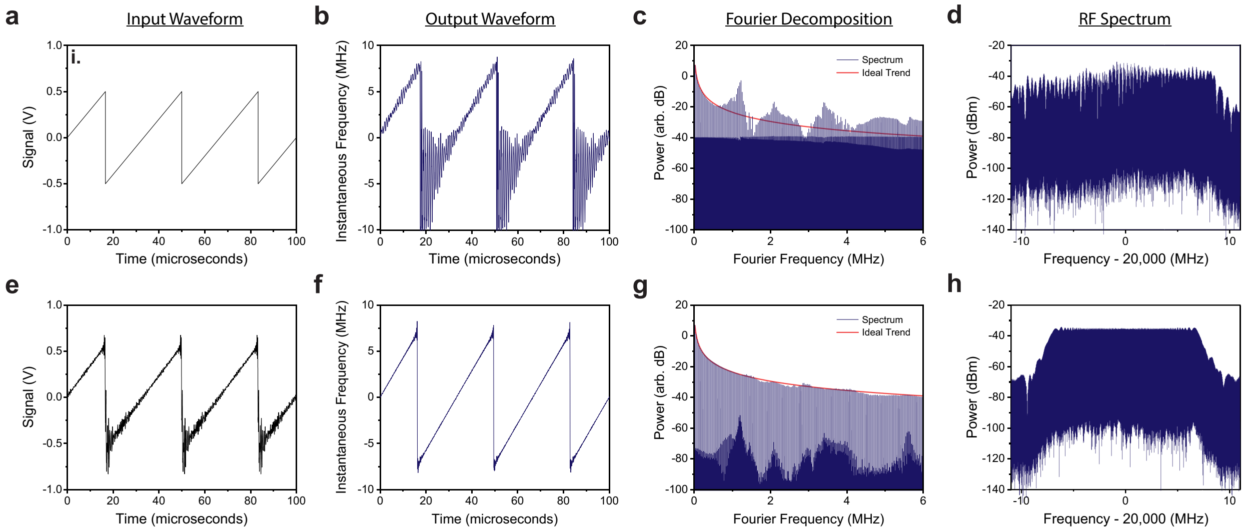

Supplementary Figure 7: Frequency modulation and response correction for a 30 kHz sawtooth wave. (a) Input voltage with amplitude of 1 V_{pp} (b) Resulting output demodulated signal, showing the sawtooth ramp FM; strong distortion is observed due to acoustic resonances in the WGMR. (c) Fourier decomposition of the output waveform, showing the power amplitude of its constituent harmonics from 0-6 MHz (blue) and the expected trend for an ideal sawtooth wave (red). (d) The RF spectrum of the modulated wave around a carrier frequency of 20 GHz. (e) Input voltage corrected according to the method outlined in the text. (f) Demodulated signal, representing vastly improved sawtooth FM. (g) Fourier decomposition of this waveform; the measured power amplitude of the various harmonics (blue) closely matches the ideal trend (red). (h) RF spectrum of the corrected FM signal.

the receiver end in a practical FM radar system.

In summary, we demonstrated direct voltage-controlled frequency tuning and modulation of our heterodyne synthesizer. Frequency modulation by a number of arbitary waveforms was achieved at modulating frequencies of 10-30 kHz, with peak frequency deviations in the range of ±5-40 MHz. These modulation frequencies and bandwidths are compatible with existing scientific FMCW radars, such as JPL's VIPR and GAISR instruments. For discontinuous and high-frequency waveforms, we showed that a pre-emphasis technique could correct distortion induced through elasto-optic FM. This form of direct optical frequency modulation is envisioned to be an important capability for a future photonics-based radar, potentially eliminating the need for an external electronic chirp synthesizer.

SUPPLEMENTARY NOTE 5: COMPARISON OF COMPACT HIGH-FREQUENCY OSCILLATORS

Here, we briefly compare the performance of our heterodyne synthesizer with a number of electronic and compact photonic frequency generators, summarized in Supplementary Table . For each source, the type, frequency range, typical voltage and power consumption, and phase noise at frequency offsets of 100 kHz and 1 MHz are specified. To provide a direct comparison in terms of noise performance between these various sources, the phase noise is also scaled to a operating frequency of 100 GHz in the rightmost columns (shaded

gray). Assuming that the multiplication/division process does not introduce additional phase noise, frequency scaling by a factor M modifies the phase noise level by $20 \log_{10}(M)$.

Notably, the noise performance of the photonic heterodyne synthesizer compares favorably with high-performance electronic synthesizers that require much higher supply power [15–17]. None of these electronic synthesizers natively support output frequencies above 90 GHz without the additional of additional frequency multiplication hardware. The heterodyne synthesizer also compares reasonably well with fixed-frequency electronic oscillators; its normalized phase noise level is superior to commercial phase-locked dielectric resonator oscillator (PLDRO) sources at frequency offsets of 100 kHz and 1 MHz [18, 19], and comes close to matching the highest-performance single-frequency sources available [20, 21] while maintaining wideband tunability.

A wide array of photonics-based sources have been studied for microwave and millimeter-wave generation. The performance of several such compact photonics-based oscillators and synthesizers is tabulated in the bottom part of Supplementary Table . Among the sources listed, the phase noise performance of the heterodyne synthesizer is surpassed only by commercial fixed-frequency opto-electronic oscillators (OEOs) operating at lower frequencies [22, 23]. Experimental work has demonstrated tunable OEOs [14, 24, 25], but implementing frequency-agility has so far come with significantly worse noise performance. More generally, while OEOs permit very low noise operation among fixed-frequency oscillators, they are intrinsically frequency-limited by their electro-optic modulators, resulting in noise performance degradation at higher frequencies. Photodiode frequency rolloff also contributes to worse noise performance at high frequencies since it represents an additional loss in the opto-electronic loop. As a result, OEOs experience similar scaling issues as electronic oscillators when translating to higher frequencies.

Microresonator-based frequency combs (MFCs) are another leading candidate for high-frequency generation and offer the potential for compact integration. In particular, the achievable close-in phase noise of MFCs can be very low. However, MFCs have essentially fixed operation frequencies set by the cavity geometry [26, 27], and their noise performance is limited by that of the pump laser, making it challenging to translate the impressive noise performance of lab-scale experiments to miniaturized devices. Notably, the performance of our synthesizer is close to that of some of the best fixed-frequency MFCs demonstrated recently [28, 29]. Looking forward, we anticipate that significant improvements for both MFCs and the self-injection-locked lasers used in our heterodyne synthesizer are possible, so that improved phase noise should be achievable in the near future.

For applications where even lower phase noise is required, photonics has enabled record-level performance, typically based on bulky mode-locked lasers [30, 31] or optical frequency division [32]. Thus far, these have power consumption in the hundreds of watts and require lab-scale setups, posing a significant challenge for integration in air- or space-borne radar instruments.

Reference	Type	Frequency	Voltage/Power Typ.	Phase Noise at 100 kHz offset (dBc/Hz)	Phase Noise at 1 MHz offset (dBc/Hz)	Phase Noise Meas. Frequency	100 GHz Scaled PN at 100 kHz offset (dBc/Hz)	100 GHz Scaled PN at 1 MHz offset (dBc/Hz)
[15] Keysight E8257D PSG Option UNY	Electronic Synth.	0.25 MHz - 67 GHz	120 VAC, 250 W	-110	-126	67 GHz	-107	-123
[16] NI QuickSyn Lite mmW FSL-7682	Electronic Synth.	76 - 82 GHz	12 VDC, 16 W	-101	-100	82 GHz	-99	-98
[17] Berkeley Nucleonics Model 865, Option LN	Electronic Synth.	0.1 MHz - 40 GHz	24 VDC, 25 W	-123	-123	20 GHz	-109	-109
[18] Polaris SPLDRO	PLDRO	26 GHz	12 VDC, 5.4 W	-110	-125	26 GHz	-98	-113
[19] Microwave Dynamics PLO-3000	PLDRO	22 GHz	12 VDC, 3.6 W	-111	-130	22 GHz	-98	-117
[20] L3Harris Narda-MITEQ LCDRO	Free Running DRO	15 GHz	5-15 VDC, 1̃ W	-139	-150	15 GHz	-122	-137
[21] Wenzel GMXO 501-27304	OCXO	12 GHz	15 VDC, 13.5 W	-137	-138	12 GHz	-119	-120
[22] OEwaves OE3700 Hi-Q(TM) Compact OEO	OEO	12 GHz	28 W	-150	-158	12 GHz	-132	-140
[23] OEwaves OE3710 Hi-Q(TM) Nano OEO	OEO	36 GHz	2.5 W	-135	-139	36 GHz	-126	-130
[14]	Tunable OEO	8-11.8 GHz	-	-119	-137	9.8 GHz	-99	-117
[24]	Tunable OEO	6.5-11.5 GHz	-	-107	-137	10 GHz	-87	-117
[25]	Tunable SBS OEO	5-40 GHz	-	-100	-120	40 GHz	-92	-112
[28]	MFC	9.9 GHz	-	-130	-148	9.9 GHz	-110	-128
[29]	MFC	19.6 GHz	-	-130	-142	19.6 GHz	-116	-128
[27]	MFC	191 GHz*	-	-82	-98	191 GHz	-88	-104
[27]	Locked MFC	191 GHz*	-	-98	-91	191 GHz	-104	-97
[33]	SBS Laser	21.7 GHz	-	-110	-125	21.7 GHz	-97	-112
This Work	Laser Heterodyne	0.5-104 GHz	6 VDC, 5.5 W	-109	-129	100 GHz	**-109**	**-129**

Supplementary Table 1: Comparison of some compact high-frequency oscillators and synthesizers. The top rows list specifications for several electronic sources, while photonic signal generators are listed in the bottom part of the table. Abbreviations: PLDRO = phase-locked dielectric resonator oscillator; DRO = dielectric resonator oscillator; OCXO = oven-controlled crystal oscillator; OEO = opto-electronic oscillator; SBS = stimulated Brillouin scattering; MFC = microresonator frequency comb; PN = phase noise. Single operating frequencies are specified for fixed oscillators, while ranges are specified for tunable synthesizers. (*) indicates that no direct output was achieved in this case due to the lack of suitable photodiode.

SUPPLEMENTARY REFERENCES

[1] Hewlett-Packard. Phase noise characterization of microwave oscillators, frequency discriminator method. *HP Product Note 11729C-2* (1985).

[2] Yao, X.S. & Maleki, L. Optoelectronic microwave oscillator. *J. Opt. Soc. Am. B* **13**, 1725–1735 (1996).

[3] Rubiola, E., Salik, E., Huang, S., Yu, N. & Maleki, L. Photonic-delay technique for phase-noise measurement of microwave oscillators. *J. Opt. Soc. Am. B* **22**, 987–997 (2005).

[4] Llopis, O., Merrer, P.H., Brahimi, H., Saleh, K. & Lacroix, P. Phase noise measurement of a narrow linewidth cw laser using delay line approaches. *Opt. Lett.* **36**, 2713–2715 (2011).

[5] Tran, M.A., Huang, D. & Bowers, J.E. Tutorial on narrow linewidth tunable semiconductor lasers using Si/III-V heterogeneous integration. *APL Photonics* **4**, 111101 (2019).

[6] Ma, L.S., Jungner, P., Ye, J. & Hall, J.L. Delivering the same optical frequency at two places: accurate cancellation of phase noise introduced by an optical fiber or other time-varying path. *Opt. Lett.* **19**, 1777–1779 (1994).

[7] Sullivan, D.B., Allan, D.W., Howe, D.A. & Walls, F.L. *Characterization of clocks and oscillators* (National Institute of Standards and Technology Technical Note 1337, 1990).

[8] Hjelme, D.R., Mickelson, A.R. & Beausoleil, R.G. Semiconductor laser stabilization by external optical feedback. *IEEE J. Quantum Electron.* **27**, 352–372 (1991).

[9] Walls, F.L. & Wainwright, A.E. Measurement of the short-term stability of quartz crystal resonators and the implications for crystal oscillator design and applications. *IEEE Trans. Instrum. Meas.* **24**, 15–20 (1975).

[10] Matei, D. *et al.* 1.5 μm lasers with sub-10 mHz linewidth. *Phys. Rev. Lett.* **118**, 263202 (2017).

[11] Beasley, P.D. The influence of transmitter phase noise on FMCW radar performance. In *2006 European Microwave Conference*, 1810–1813 (IEEE, 2006).

[12] Siddiq, K., Hobden, M.K., Pennock, S.R. & Watson, R.J. Phase noise in FMCW radar systems. *IEEE Trans. Aerosp. Electron. Syst.* **55**, 70–81 (2018).

[13] Liang, W., Eliyahu, D., Savchenkov, A. & Matsko, A. A low-RIN spectrally pure whispering-gallery-mode resonator-based semiconductor laser. *IEEE Photon. Technol. Lett.* **30**, 1933–1936 (2018).

[14] Savchenkov, A.A. *et al.* Voltage-controlled photonic oscillator. *Opt. Lett.* **35**, 1572–1574 (2010).

[15] Keysight Technologies. *E8257D PSG Microwave Analog Signal Generator* (2020). URL `https://www.keysight.com/us/en/assets/7018-01211/data-sheets/5989-0698.pdf`.

[16] National Instruments. *QuickSyn Frequency Synthesizers* (2019). URL `http://ni-microwavecomponents.com/datasheets/QuickSyn_Brochure_Datasheet.pdf`.

[17] National Instruments. *Model 865 RF / Ultra Low Noise Microwave Signal Generator* (2020). URL `https://www.berkeleynucleonics.com/sites/default/files/products/datasheets/865_datasheet_v1.33_2020_10_20-jl.pdf`.

[18] Polaris. *Single Loop PLDRO* (2019). URL `http://polariswave.com/wp-content/uploads/2019/12/Single-Loop-PLDRO-rev4.0.pdf`.

[19] Microwave Dynamics. *Internal Reference PLDRO (PLO-3000)* (2016). URL `http://microwave-dynamics.com/wp-content/uploads/2016/05/PLO-3000-1.pdf`.

[20] L3Harris Narda-MITEQ. *LCDRO Series* (2019). URL https://nardamiteq.com/docs/L3Harris_LCDRO_datasheet.pdf.

[21] Wenzel Associates. *12.0 GHz Golden Multiplied Crystal Oscillator (GMXO-FR)* (2014). URL https://wenzel.com/wp-content/parts/501-27304.pdf.

[22] OEwaves. *HI-Q™ Compact Opto-electronic Oscillator* (2019). URL https://www.oewaves.com/oe3700.

[23] OEwaves. *HI-Q™ Nano Opto-electronic Oscillator* (2019). URL https://www.oewaves.com/oe3710.

[24] Li, W. & Yao, J. An optically tunable optoelectronic oscillator. *J. Light. Technol.* **28**, 2640–2645 (2010).

[25] Merklein, M. *et al.* Widely tunable, low phase noise microwave source based on a photonic chip. *Opt. Lett.* **41**, 4633–4636 (2016).

[26] Xue, X. *et al.* Thermal tuning of Kerr frequency combs in silicon nitride microring resonators. *Opt. Express* **24**, 687–698 (2016).

[27] Liu, J. *et al.* Monolithic piezoelectric control of soliton microcombs. *Nature* **583**, 385–390 (2020).

[28] Liang, W. *et al.* High spectral purity Kerr frequency comb radio frequency photonic oscillator. *Nat. Commun.* **6**, 7957 (2015).

[29] Liu, J. *et al.* Photonic microwave generation in the X- and K-band using integrated soliton microcombs. *Nat. Photonics* **14**, 486–491 (2020).

[30] Fortier, T.M. *et al.* Generation of ultrastable microwaves via optical frequency division. *Nature Photonics* **5**, 425 (2011).

[31] Xie, X. *et al.* Photonic microwave signals with zeptosecond-level absolute timing noise. *Nature Photonics* **11**, 44 (2017).

[32] Li, J., Yi, X., Lee, H., Diddams, S.A. & Vahala, K.J. Electro-optical frequency division and stable microwave synthesis. *Science* **345**, 309–313 (2014).

[33] Li, J., Lee, H. & Vahala, K.J. Microwave synthesizer using an on-chip Brillouin oscillator. *Nat. Commun.* **4**, 2097 (2013).
